# ChiRA: an integrated framework for chimeric read analysis from RNA-RNA interactome and RNA structurome data

**DOI:** 10.1093/gigascience/giaa158

**Published:** 2021-01-29

**Authors:** Pavankumar Videm, Anup Kumar, Oleg Zharkov, Björn Andreas Grüning, Rolf Backofen

**Affiliations:** Bioinformatics Group, Department of Computer Science, University of Freiburg, Georges-Koehler-Allee 106, 79110 Freiburg, Germany; Bioinformatics Group, Department of Computer Science, University of Freiburg, Georges-Koehler-Allee 106, 79110 Freiburg, Germany; Bioinformatics Group, Department of Computer Science, University of Freiburg, Georges-Koehler-Allee 106, 79110 Freiburg, Germany; Bioinformatics Group, Department of Computer Science, University of Freiburg, Georges-Koehler-Allee 106, 79110 Freiburg, Germany; Bioinformatics Group, Department of Computer Science, University of Freiburg, Georges-Koehler-Allee 106, 79110 Freiburg, Germany; Signalling Research Centres BIOSS and CIBSS, University of Freiburg, Schaenzlestr. 18, 79104 Freiburg, Germany

**Keywords:** microRNA, chimeric read, RNA-RNA interactome, structurome, visualization, CLASH, CLEAR-CLIP, PARIS, SPLASH, Galaxy workflow

## Abstract

**Background:**

With the advances in next-generation sequencing technologies, it is possible to determine RNA-RNA interaction and RNA structure predictions on a genome-wide level. The reads from these experiments usually are chimeric, with each arm generated from one of the interaction partners. Owing to short read lengths, often these sequenced arms ambiguously map to multiple locations. Thus, inferring the origin of these can be quite complicated. Here we present ChiRA, a generic framework for sensitive annotation of these chimeric reads, which in turn can be used to predict the sequenced hybrids.

**Results:**

Grouping reference loci on the basis of aligned common reads and quantification improved the handling of the multi-mapped reads in contrast to common strategies such as the selection of the longest hit or a random choice among all hits. On benchmark data ChiRA improved the number of correct alignments to the reference up to 3-fold. It is shown that the genes that belong to the common read loci share the same protein families or similar pathways. In published data, ChiRA could detect 3 times more new interactions compared to existing approaches. In addition, ChiRAViz can be used to visualize and filter large chimeric datasets intuitively.

**Conclusion:**

ChiRA tool suite provides a complete analysis and visualization framework along with ready-to-use Galaxy workflows and tutorials for RNA-RNA interactome and structurome datasets. Common read loci built by ChiRA can rescue multi-mapped reads on paralogous genes without requiring any information on gene relations. We showed that ChiRA is sensitive in detecting new RNA-RNA interactions from published RNA-RNA interactome datasets.

## Introduction

Many non-coding RNAs (ncRNAs) regulate gene expression, post-transcriptionally, via mechanisms such as activation or inhibition of translation, destabilization, localization, and processing. For example, a microRNA (miRNA) can downregulate target expression via translational inhibition or transcript destabilization, initiated by the formation of base pairs between the mature miRNA (~22 nt long) and the target RNA transcript [1]. For successful regulation, not only the intermolecular structure (i.e., the RNA-RNA interaction) but also the structure of the ncRNA itself (i.e., the intramolecular RNA structure) is key to the regulatory process [2–4] because it influences the parts of the ncRNA that are accessible for RNA-RNA interactions. Computationally, the prediction of both inter- and intramolecular structure is non-trivial and results can be unreliable [5]. To support computational methods, several transcriptome-wide experimental protocols have been developed recently to detect both inter- and intramolecular RNA structure [6–10]. Although these protocols vary in their application-specific details, they currently all involve ligating the 2 RNA interaction partners together and subsequently sequencing the resulting chimeric RNA molecules using high-throughput sequencing technology. Chimeric RNAs from gene fusions by trans-splicing or chromosomal rearrangements can also be seen in RNA sequencing data. Such chimeric RNAs are often associated with specific cancer types [11,12] and considered to be potential biomarkers [13,14].

MicroRNAs have been a subject of avid research in the past decade owing mostly to 2 reasons: (i) it is proposed that each miRNA can regulate up to several hundred targets and that a substantial proportion of protein-coding genes are targeted by miRNAs at some stage [15] and (ii) individual miRNAs have been implicated in several notorious human diseases, such as different cancer types and neurodegenerative illnesses [16–18]. Therefore, accurate identification of miRNA targets is highly sought after. Despite numerous attempts, computational prediction approaches still deliver poor results with generally high false-positive rates, with no significant improvement observed in the past decade (see review [19]). Therefore, considerable effort has also gone into developing high-throughput experimental protocols, specifically designed to detect miRNA-target interactions (reviewed in [20]). The most recent line of development has been to ligate the miRNA to the site-specific interaction region of the target, selecting these interactions via cross-linking to 1 of the Argonaute proteins required for miRNA-based regulation, and to sequence the resulting chimeric RNA molecule, e.g., CLASH [6] and CLEAR-CLIP protocols [7]. Going beyond miRNAs, these protocols can obviously be applied to RNA interactions that involve a regulatory protein other than Argonaute. To generalize even further, researchers have applied the same idea to the detection of all transcriptome-wide RNA-RNA interactions. This includes both inter- and intramolecular base-pairing without the necessity of choosing a specific regulatory protein for cross-linking, as done, e.g., in PARIS [8], SPLASH [9], and LIGR-Seq [10]. Regardless of the protocol, the sequenced reads are chimeric; i.e., a fusion of 2 different RNA fragments corresponds either to intermolecular interaction or to 2 distinct parts of a single RNA molecule from its intramolecular structure.

Two main computational challenges arise from such chimeric-read data: (i) mapping the chimeric reads to 2 different locations on reference transcript annotations and (ii) dealing with the fact that these short RNA segments map to multiple locations, i.e., specifically dealing with multi-mapped reads. State-of-the-art mapping software, such as Bowtie2 [21], BWA-MEM [22], and STAR [23], can both map chimeric reads and allow for multiple mapping locations, given the appropriate parameter settings. Subsequent to mapping, however, there are no satisfactory or standard solutions for correctly quantifying multi-mapped reads. Multi-mapped reads are either ignored or incorrectly assigned and/or quantified. Three common approaches exist for assigning multi-mapped reads: (i) they are not assigned but simply discarded; (ii) a read is assigned to each of the multi-mapped locations with equal distribution (e.g., with a count of 1 divided by the number of locations); and (iii) the true expression level is estimated by assigning the read to a multi-mapped location proportionally to the number of uniquely mapped reads in the vicinity of that location. The ability of the resulting read counts to capture expression levels or RNA interaction events increases with each approach. Obviously, discarding multi-mapped reads is a poor solution and definitely not an option when dealing with chimeric reads. Distributing counts equally under- or overestimates the actual expression in all locations in comparison to regions with uniquely mapping reads. The third approach can deliver accurate results but fails when it comes to distributing reads among gene families with very similar sequences, e.g., for miRNA gene families.

Existing software solutions that take the raw data input from RNA-interactome protocols and deliver quality interaction annotations are currently application or protocol specific. Most of them were released along with their corresponding published experimental protocols, and none of them has become a readily usable bioinformatic pipeline. There also exist generic standalone pipelines like Hyb [24], which was developed and demonstrated to deal with miRNA-specific data. From the computational side, there is thus still a major hurdle to overcome before such protocols can be broadly applied in practice: the availability of easy-to-use software that can process the raw data to produce accurate annotation and quantification of the identified RNA-RNA interactions. Here we present a method to resolve multi-mapping to very similar reference sequences from possible gene families and paralogs without requiring any prior annotation. Our method determines the best alignment for each multi-mapped read by an elegant quantification and scores them on the basis of the abundance of reference loci. Our ChiRA tool suite, Galaxy [25] workflows, and visualization provide a complete analysis framework for chimeric reads from RNA-RNA interactome and RNA structurome protocols. Thus, we aim to strengthen a weak link in the search for transcriptome-wide RNA interactions/structures.

## Methods

**Figure 1:**
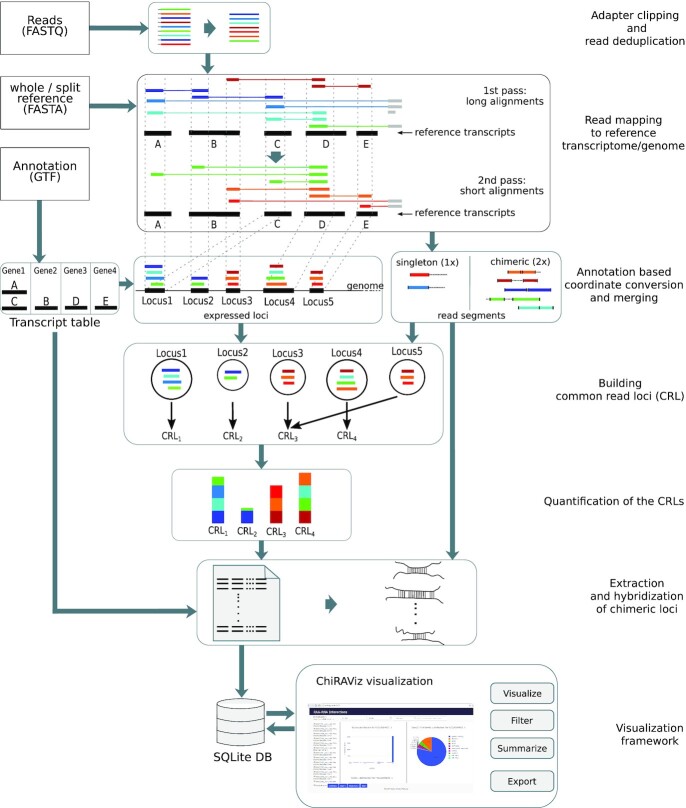
ChiRA workflow. First, the reads are deduplicated and mapped to reference sequences. Then the overlapping reference regions are merged into expressed loci. Given an annotation file, transcriptomic alignment positions are converted into genomic positions. Common read loci are built on the basis of the reads that are consistently multi-mapped among the expressed loci. The quantification is carried out at the common read loci level, and the interactions are scored and hybridized. With the visualization, users can search, filter, and export preferred interactions. Here transcripts A and C are 2 isoforms of a gene Gene1. Because of shared exons, multi-mappings to A and C can be collapsed into a single genomic locus Locus1. As Locus3 and Locus5 share all their read segments, they are merged into a single CRL. Owing to the quantification based on expectation maximization, the multi-mapped green read segment is counted more towards CRL_1_ than CRL_2_.

We built a complete workflow that takes raw sequencing reads as input and outputs a comprehensive list of annotated interacting regions. This involves read deduplication, mapping, quantification (including multiple mapped reads) of reference loci to infer the correct locations on the basis of their expression, and hybridization of interacting reference loci. To offer a convenient interface on top of ChiRA output an interactive visualization, ChiRAViz, was developed. Fig. 1 shows the complete workflow built from the ChiRA and ChiRAViz tool suite. Each of the following sections corresponds to the steps represented (listed on the right side) in the figure.

### Adapter clipping and read deduplication

Quality and adapter trimming, in general, are crucial for RNA-RNA interactome data but essential for small RNA-related interactome data. Mature miRNAs that interact with the targets are only ∼18–22 nt in length. Depending on the captured target sequence, chimeric reads often have adapters in them. In our analysis, ≥80% of sequenced reads from CLASH and CLEAR-CLIP datasets contained adapters. For our analysis we trimmed low-quality ends and adapters from the reads using cutadapt [26]. Reads that were shorter than 10 nucleotides were discarded, and the remaining reads were deduplicated to eliminate possible PCR duplicates. In general, not all identical reads are PCR duplicates. Gene isoforms or gene paralogs also result in duplicate RNA fragments. To uniquely identify the RNA fragments, unique molecular identifiers (UMIs) are used. UMIs are short sequences of a specific length that are usually attached at the 5′ end of the RNA fragments during library preparation. We also deduplicate reads based on UMIs if they are present in the library. We consider identical reads with the same UMI as PCR duplicates, whereas identical reads with different UMIs are considered unique. The deduplication step may reduce the number of reads by orders of magnitude, which in turn can speed up the subsequent steps.

### Read mapping to reference transcriptome/genome

In this step, we align the reads to the reference transcriptome or genome. For well-annotated organisms, we recommend using the transcriptome for the following reasons. (i) When mapped against a transcriptome, reads can be mapped linearly across the splice junctions. Especially, in the case of these small read fragments, it can be extremely difficult to map across the splice junctions when mapped to the genome. (ii) There is less chance of getting random false-positive hits for short read fragments on transcriptome than on whole genome. Unfortunately, except for some model organisms, reference annotations are either incomplete or unreliable. In that case, using the whole genome sequence as a reference is a good choice for the following reasons. (i) An unreliable annotation leads to false conclusions on the type of detected interactions. (ii) An incomplete annotation results in false-negative interactions. Consider an example of CLASH data that predominantly contain miRNA and 3′ untranslated region (UTR) interactions. Mapping to a reference transcriptome with an incomplete 3′ UTR annotation fails to capture the most important category of interactions.

Currently, we support mapping with BWA-MEM [22] and CLAN [27]. CLAN is a recent exclusive chimeric read mapper and outputs the chimeric alignments in tabular format. BWA-MEM is also capable of producing chimeric reads by local alignment. With a high dynamic range in read lengths, it is not always possible to accurately map chimeric reads of different lengths with a single parameter setting. Hence, when BWA-MEM is used as the aligner, we do a 2-pass alignment. The first pass targets mapping long chimeric read segments whereas the second pass targets short ones. In the first pass, we use high alignment score thresholds and allow gaps and hence achieve long gapped chimeric alignments. In the second pass, we use a lower alignment score cut-off and do not allow any insertions or deletions. Therefore the second pass rescues short chimeric read segments with perfect matches on the reference. The default alignment settings were optimized on the miRNA interactome data from CLASH and CLEAR-CLIP protocols. The complete list of alignment settings can be found in the provided Galaxy histories (see Supplementary Section S4 for more details). BWA-MEM can output the alignments in Sequence Alignment/Map (SAM) format. We convert it into Binary sequence Alignment/Map (BAM) and use pysam [28] for further processing. It is important to consider that BWA-MEM randomly chooses 1 of the alignments as primary and writes all the alternative hits to the XA tag of the alignment. The true alignment can also be hidden under the XA tag and buried in the BAM file. BWA-MEM has an option (-h) that controls the writing of these suboptimal alignments to the output BAM file. In the second pass, we set it to a high number (default 100) so that we do not miss any of the equally good alternative alignments. The idea is to get as many multi-hits as possible and let ChiRA pick the best one in subsequent steps. In the end, we combine the alignments from both the alignment steps, parse the BAM file using pysam, and write them to a Browser Extensible Data (BED) file. In this step, we only keep the alignments that are mapped on the sense reference strand. If there is an XA tag for an alignment, we keep all the alternative alignments with the highest read coverage. In the end, we remove any duplicate hits in the second pass of the 2-pass alignment.

Because each chimeric read often contains 2 RNA fragments originating from 2 different RNA types, we allow mapping to 2 different reference transcriptomes ("split reference"). For example, for CLASH data, we encourage the use of a split reference, one containing miRNAs and the other containing the rest of the transcriptome, which restricts the output to miRNA-based interactions. The parameters such as seed lengths and alignment scores are dependent on the type of the data or expected length of chimeric arms. In our experience, the default settings work well with the miRNA interactome data.

### Annotation-based coordinate conversion and merging

Given an annotation file in Gene Transfer Format (GTF), we convert transcriptome locations to genomic locations because working on the genomic locations is less ambiguous. The main problem with transcript locations is that the reads mapped to the exons that are shared among the isoforms appear to be multi-mapped. But at the genomic level, these are uniquely mapped. In the absence of a GTF file ChiRA can still work with transcriptome locations.

#### Merge reference positions to define interaction sites

Because the experimental protocols may generate several reads covering different parts of an interaction site, we have to define an interaction site by combining overlapping alignments. This step separates alignments stemming from the same interaction sites from alignments that cover a completely different interaction site on the same transcript. For example, 2 different miRNAs may target a single mRNA at 2 different locations such as coding sequence and 3′ UTR. In more detail, we merge the significantly overlapping alignments based on the reference mapping locations to generate so-called "expressed loci." A single transcript may have multiple such expressed loci. For an alignment to merge into an existing expressed locus, both the alignment and the locus must reciprocally overlap >70% (default value) in length.

While this approach works well with interaction sites that have a low to medium coverage, it might fail in the case of sites with high coverage because the likelihood of finding 2 alignments with 70% overlap at random increases. For this purpose, we have an alternative merging mechanism using blockbuster [29]. blockbuster defines the blocks of alignments based on a Gaussian approximation of the read coverage. Subsequently on the basis of the -distance parameter, it places adjacent read blocks into clusters. However, we ignore this cluster information and work further on the block level. We merge any overlapping blocks to define potential interaction loci. This approach is thus similar to (but also simpler than) the one introduced and successfully applied for cross-linking immunoprecipitation sequencing (CLIP-seq) peak calling in Holmqvist et al. [30].

#### Merge read positions to define chimeric arms

In this step, we identify all chimeric and non-chimeric (singleton) aligned reads. A chimeric read has ≥2 non-overlapping portions on the read mapped to distinct reference loci. If a sequenced read is chimeric and it is uniquely mapped to the reference, then we have ≤2 alignments each belonging to 1 chimeric arm. If a sequenced read is a singleton and mapped uniquely, then we have maximally 1 alignment. We call each aligned portion of the read a "read segment." In later steps, during quantification, a singleton read will be treated as 1 read whereas a chimeric read will be treated as 2 (1 for each segment) separate reads. Hence it is crucial to define the chimeric split points of the reads. A chimeric split point can be identified by its non-overlapping segments. Owing to local alignment and repetitive parts on the reference sequences, some overlapping segments multi-map with few bases shifted. Considering each such highly conserved read segment separately penalizes the overall read segment contribution in quantification. Hence, we further merge read segments that overlap ≥70% (default value) of their length into a single segment. In theory, there are only 2 interacting read segments because there are maximally 2 interacting RNA fragments captured in the interactome experiments. Owing to sensitive alignment settings, some reads also result in >2 segments. After a subsequent quantification step, only the 2 most probable chimeric arms will be considered for each read.

### Building common read loci

There are cases where read segments map to the gene families or paralogous loci sharing the common sequences. It is huge a challenge to find a decent annotation that carries gene family or paralog information. It was shown by Robert and Watson [31] that grouping of genes based on multi-mapped reads resulted in groups of gene families and analyzing the RNA-seq data at this group level was biologically relevant. Similarly, we propose a method to group multi-mapped loci that does not depend on any annotation. If 2 loci share a large portion of their multi-mapped reads, their sequences tend to be very similar or originate from the same gene families or paralogs or have similar pathways (see Results and Discussion). Hence, we group expressed loci into common read loci (CRL) if they share a significant number of multi-mapped reads. Here we use single-linkage clustering with the Jaccard index to measure the similarity between the expressed loci. To merge an expressed locus into an existing CRL, the Jaccard index of sets of reads between that locus and the CRL should be greater than a user-defined threshold (default of 0.7). We merge the loci in order by size. If a locus failed to share a significant portion of multi-mapped reads with any other CRL, then it gets its own CRL. If the reads were mapped to transcriptome and the user does not provide any gene annotation file, CRLs are well capable of grouping multi-mapped reads that map to gene isoforms. See Algorithm 1 for CRL creation pseudo code.

**Algorithm 1:**
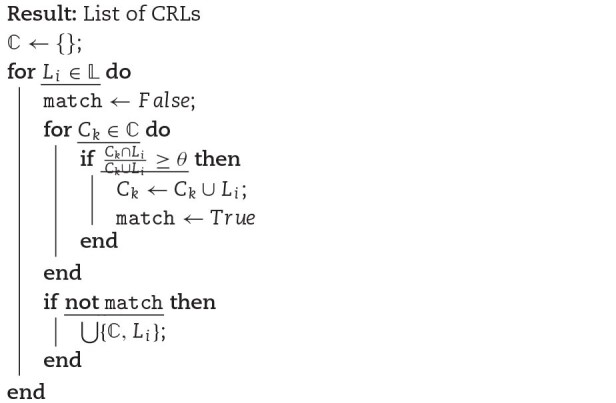
CRL creation from expressed loci. $\mathbb{C}$ is the list of CRLs; $\mathbb{L}$ is the list of expressed loci; $L_i$ is the set of read segments of an expressed locus *i* and $C_k$ is the set of read segments of a CRL *k*.

### Quantification of the CRLs

To score the mapped chimeric reads, we first need to estimate the expression of the CRLs by quantification. Quantification helps to assess the true origin of a read segment in the case of multi-mapping. It has been shown that proper quantification of multi-mapped reads led to the discovery of novel protein-RNA interactions from CLIP-seq data  [32,33]. A study on RNA-seq data revealed that the expression of genes with multi-mapped reads was underestimated by common quantification methods [31]. There exist comprehensive studies on methods [34,35] and metrics [36] for quantification of RNA-seq data, but direct application of these methods to our data is not possible for the following reasons. First, it is hard to supply our pre-built locus-CRL relations to the quantification tools on the fly. Second, unlike our short reference loci, the reference RNAs in RNA-seq have multiple exons and are much longer. In RNA-seq, often the quantification is done at the isoform level, where exons that are unique to that isoform help to resolve the multi-mapping by estimating the total maximum likelihood for that isoform. But in interactome data, there is only a part of the interacting exons that is captured and the rest is missing. If this interacting part of an exon is shared among the isoforms, the read segments mapped are still called multi-mapped and each transcript gets an equal share from the read segment. Therefore we implemented an approach to quantify the CRLs based on the expectation-maximization (EM) algorithm. In this quantification, all multi-mapped reads that map to different expressed loci of a CRL are considered as uniquely mapped to that CRL.

Let $\mathbb {S}$ be the set of all read segments with $N=|\mathbb {S}|$ and $\mathbb {C}$ be the set of all CRLs with $K=|\mathbb {C}|$. We follow Xing et al. [37] in the annotation, where we estimate the CRL abundance by determining the likelihood ρ_*c*_ = Pr[*s* ∈ *c*] that a read segment *s* actually stemmed from CRL *c*. We denote with ρ the vector of all ρ_*c*_. Note that when the CRLs have a similar length as in our case, length normalization can be omitted; i.e., ρ_*c*_ are then direct estimates for CRL abundances. In the case of multiple mapping, we define 2 indicator variable matrices to model the read segment selection process. We have an *N* × *K* indicator matrix $Z=(\rm z_{s,c})_{{{_{\rm c\,\,\in\,\,\mathbb {C}}^{\rm s\,\,\in\,\,\mathbb {S}}}}}$ with

\begin{equation*} z_{s,c} = \left\lbrace \begin{array}{@{}l@{\quad }l@{}}1 & \text{if read segment}\ s\ \text{is from CRL}\ c\\ 0 & \text{else} \end{array}\right. \end{equation*}

However, this is not directly observable in the case that the reads map to different CRLs. This can be overcome by introducing another matrix $Y=(y_{s,c})_{{{_{\rm c\,\,\in\,\,\mathbb {C}}^{\rm s\,\,\in\,\,\mathbb {S}}}}}$ with

\begin{equation*} y_{s,c} = \left\lbrace \begin{array}{@{}l@{\quad }l@{}}1 & \text{if read segment}\ s\ \text{maps to CRL}\ c\\ 0 & \text{else} \end{array}\right. \end{equation*}

Note that we have in each row of *Z* exactly 1 entry with 1, whereas in *Y* we can have several such entries. Furthermore, *y_s,c_* = 0 implies *z_s,c_* = 0. We call *Z* the committed categorization and *Y* the uncommitted categorization. In the case of multiple mappings, we have many different *Z*-matrices that are compatible with *Y* (meaning that each row in *Z* has sum 1, and *y_s,c_* = 0 implies *z_s,c_* = 0) and are unobservable. Then, the likelihood of the observation (i.e., read segments) ${\cal L}(\rho )$ is defined as follows:

\begin{equation*} {\cal L}(\rho )=\prod _s\sum _{c} y_{s,c}\rho _{c}. \end{equation*}

However, this maximum likelihood solution for ${\cal L}(\rho )$ cannot be obtained in closed form. Hence, we apply the following EM algorithm to determine the maximal likelihood estimates $\hat{\rho }$.

#### E-Step

Let ρ^(*t*)^ be the vector of abundance estimates $\rho ^{(t)}_c$ in round *t* of the EM algorithm. The E-Step consists of the determination of the expected values for the hidden variables:

(1) \begin{eqnarray*}
E\left[z_{s,c}\mid Y,\rho ^{(t)}\right] &=& \mathrm{Pr}\left(z_{s,c}=1\mid \rho ^{(t)},Y\right)\nonumber \\ &=& \dfrac {\rho ^{(t)}_c}{ \sum _{c^{\prime }} y_{s,c^{\prime }}\rho ^{(t)}_{c^{\prime }}}. \end{eqnarray*}

Note that we are interested not only in determining the abundances of the CRLs but also in the likelihood that a read segment *s* is from a CRL *c*, i.e., in $\mathrm{Pr}(z_{s,c}=1\mid \hat{\rho },Y)$, for which we can use the values calculated in equation (1) in the last E-Step of the EM algorithm. From these likelihoods, we can calculate the probability Pr[(*s, s*′) ∈ *c*↔*c*′] that a chimeric read ..*s*..*s*′.. is an interaction between CRLs *c* and *c*′:

\begin{equation*} \mathrm{Pr}[(s,s^{\prime })\in c\leftrightarrow c^{\prime }]= \mathrm{Pr}(z_{s,c}=1\mid \hat{\rho },Y)\mathrm{Pr}(z_{s^{\prime },c^{\prime }}=1\mid \hat{\rho },Y). \end{equation*}

Note that the relative abundance of the transcript does not influence this probability because we consider only the read segment *s* (respectively *s*′) and $\sum _{c}y_{s,c}\mathrm{Pr}(z_{s,c}=1\mid \hat{\rho },Y)=1$ [respectively $\sum _{c}y_{s^{\prime },c}\mathrm{Pr}(z_{s^{\prime },c}=1\mid \hat{\rho },Y)=1$].

#### M-Step

The M-Step is simply the maximum likelihood estimate, given the hidden values *z*:

(2) \begin{eqnarray*}
\rho ^{(t+1)}_c = \frac{\sum _{s}z^{(t+1)}_{s,c}}{N}. \end{eqnarray*}

We repeat the E and M steps until the sum of differences between the relative abundances of CRLs in 2 consecutive iterations is not higher than a user-defined value ϵ, i.e., $\sum _{c=1}^K |\rho _c^{(t+1)} - \rho _c^t| \le \epsilon$. The default value for ϵ that we use is 1*e*^−5^. The expression levels of the CRLs are reported in transcripts per million (TPM). Calculation of TPM is explained in Supplementary Section S3.

### Extraction and hybridization of chimeric loci

In this final step, we extract the 2 most probable chimeric arms for each chimeric read along with their alignment and sequence information. If a GTF file is provided, we annotate the interacting regions with gene IDs, symbols, biotypes, and so forth. For protein-coding genes, the biotypes are further categorized into 5′ UTR, coding sequence, and 3′ UTR. For hybridization of chimeric arms we use IntaRNA [38]. Occasionally, the real interaction is in the vicinity of the sequenced arms. For this reason, we hybridize the reference loci sequences from the output instead of the aligned read sequences. These reference loci are merged from multiple overlapping alignments and already contain some context of mapped arm locations.

### Visualization framework

#### Motivation

ChiRAViz visualizer is developed in JavaScript (JS) to summarize, filter, and visualize the output of ChiRA. The output of ChiRA is a tabular file with each record containing interacting positions of a read on the reference with their annotation information (in case GTF was provided during the analysis) such as gene IDs, biotypes, gene symbols, alignment information, and so fort. Each such record contains >30 columns, and depending on the library size and the complexity of the interactome there can be millions of records in a single output file. Working with such large data is hard, especially extracting elements of significant interactions from their native tabular form. Therefore, to summarize the complete data, a visualizer is needed where information can be filtered and shown in the form of various charts that are easier to understand.

#### Datatype

The visualizer is integrated into Galaxy as a native visualization for chira.sqlite datatype. Using a database allows SQLite queries to be formulated and executed to fetch a subset of data by applying filters on its columns.

#### User Interface

The user interface (UI) of the visualizer is created using JS and multiple JS-related packages such as UnderscoreJS, Bootstrap, and jQuery. UnderscoreJS methods are used for better manipulation of JS arrays and dictionaries. Bootstrap is used for styling the UI and jQuery for document object model manipulation and asynchronous methods to fetch data from the database file.

## Results and Discussion

### Data

We applied ChiRA on a custom-made benchmark dataset to assess the performance, and on published RNA-RNA interactome and structurome datasets to validate the approach and showcase the functionality.

#### Benchmark data

Based on the benchmark data provided by the CLAN publication, we produced our benchmark data to test the performance of ChiRA. The reads were unchanged, but we modified the reference sequences. The reads imitate CLASH experimental data. Each read is a direct fusion of (sub)sequences of human hg38 miRBase [39] mature miRNAs and a random TargetScan [40] target sequence (i.e., the target sequence is not necessarily a true target of this miRNA). The reads are in FASTA format and contain 1 million reads per sample. There are 5 different samples of simulated chimeric reads, each containing a specific chimeric arm length (10, 12, 15, 18, and 20). These datasets are called "noInsert" data. There is a second set of data with the same arm lengths but a random 5-nucleotide sequence inserted either between or at the ends of the arms of each chimeric read. This dataset is called "Insert" data. In both cases, if the reference miRNA or reference TargetScan target is shorter than the arm length, the whole reference sequence was used.

As a reference database, we used miRBase mature miRNAs together with TargetScan target sites. The reference sequences used in the CLAN publication were very short in length, with a mean length of 21 nt for miRNAs and 14 nt for target reference sequences. Using those short TargetScan targets only as a reference is not realistic. Moreover, the TargetScan target sequences were predicted by a computational approach and generally not used as a reference database. With very short target sequences it is fairly easy for the aligners to map the reads to exact locations uniquely. Adding some context poses an additional challenge to the aligners and results in multi- or wrong alignments. Hence, to test the potential of our workflow on more complicated and near real-world reference sequences, we modified the target reference data as follows. First, we sorted all the target genomic regions and then extended each region until the next target region was within a 200-nt range. In the end, we extracted the sequences of these positions. This procedure results in target sequences of various lengths. Similar to the real reference database, there is also a fair chance of having multiple target sites on a single reference sequence. In the original CLAN benchmark data, there were duplicate reference sequences. These were coming from the same duplicated targets of different miRNAs. All these duplicated reference sequences have been removed from our benchmark data.

#### Published data

To show the functionality of ChiRA, we applied ChiRA also on published datasets. We analyzed human miRNA interactome data from CLASH and mouse interactome data from CLEAR-CLIP protocols. For RNA-RNA interactome and structurome data, we used polyA enriched SPLASH samples from lymphoblastoid cells, human embryonic stem cells (ES), and human retinoic acid differentiated cells, as well as mouse ES and human HEK293T samples from PARIS protocol. For CLASH and CLEAR-CLIP we built the reference databases as explained in the methods from their respective articles. For SPLASH and PARIS datasets we used the complementary DNA sequences of hg38 and mm10 genome builds from Ensembl revision 100. A summary of published data and their processing is provided in the Supplementary Section S2.

### Performance on the benchmark data

**Figure 2:**
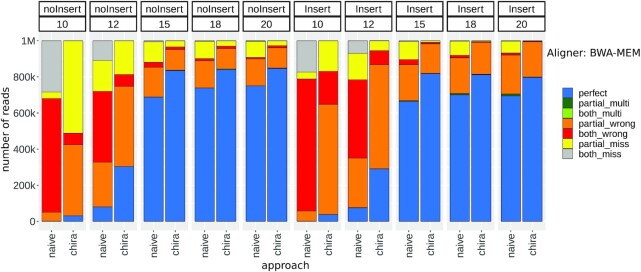
Performance of BWA-MEM–based ChiRA compared to naive approach on benchmark data. ChiRA-based results have ≥10% more perfect hits compared to naive mode for any arm length.

**Figure 3:**
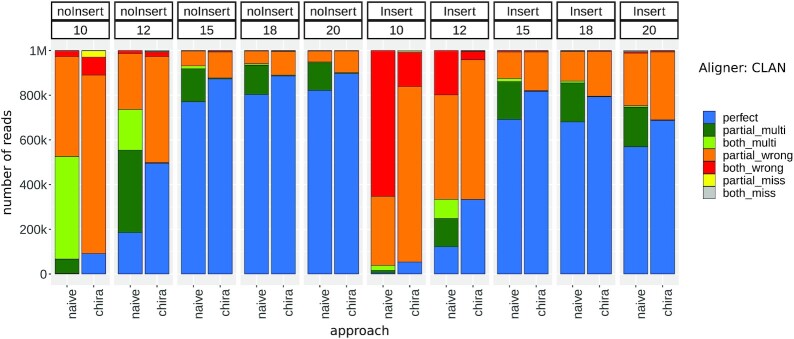
Performance of CLAN-based ChiRA compared to naive approach on benchmark data. Being a chimeric read aligner CLAN produced fewer wrong hits and more multi-mapped hits that contain the true alignment. CRL-based ChiRA could pick the correct reference from the multi-mapped hits for any arm length. Note that although there are more multi-hits (green) in naive mode compared to chira mode, the origin of these reads is still uncertain.

We chose the same terminology as in the CLAN article to categorize the reads on the basis of alignment types. An "arm" is an arm of chimeric read segments, and an "agreed arm" is an arm that has an alignment with ≥80% overlap on correct reference location. The categories are defined as follows: "perfect": has both uniquely mapped agreed arms; "partial_multi": has 1 uniquely mapped agreed arm and 1 multi-mapped agreed arm; "both_multi": both arms are multi-mapped agreed arms; "partial_wrong": has 1 uniquely mapped agreed arm and 1 wrongly mapped; "both_wrong": both arms are wrongly mapped; "partial_miss": has 1 mapped and 1 unmapped arm; "both_miss": both arms are unmapped. We carried out 2 separate runs of ChiRA using BWA-MEM and CLAN aligners. Figs. 2 and 3 show their respective performance. Each bar in the plot represents the result of 1 of the 2 modes "naive" or "chira." The naive mode involves running the alignment tool (BWA-MEM or CLAN depending on the run) on the single reference database obtained by concatenating both mature miRNAs and TargetScan targets together, resulting in a gapped alignment. The reads are then directly categorized into 1 of the 7 aforementioned categories. When using BWA-MEM in naive mode, we considered only the longest alignment for each arm. In cases of multiple longest alignments, we considered all of them. In the chira mode, the ChiRA workflow with the corresponding aligner was used to obtain the results. In this mode, we used a split reference, i.e., the 2 separate reference databases for mature miRNAs and target sequences. We also enabled CRL creation while quantifying. The bars are then grouped horizontally on the basis of the arm lengths and then furthermore grouped by whether the reads contain inserts.

The most challenging cases are with arm lengths of 10 and 12 nt. Being very short sequences, these cases tend to result in a lot more multi-mappings than the others. In naive mode, for an arm length of 10 nt there are a negligible number of perfect reads. The chira mode could detect some perfect reads, but they are still <10% in any case. Considering the short length of the arms, it is clear that these generally map to multiple or wrong locations. For an arm length of 12 nt, there is >2.5-fold increment in perfect reads from naive to chira mode. At this arm length there is still not an acceptable number of perfect reads except for the CLAN aligner on noInsert data. The percentages of perfect reads are consistently ∼70% for arms of lengths ≥15 nt for both the aligners in naive mode. This observation indicates that the sequenced RNA fragments must be ≥15 nt long to be uniquely identified at an acceptable rate. Despite being a chimeric read aligner, CLAN produced a significant amount of ambiguous partial_multi and both_multi alignments in naive mode (Fig. 3). ChiRA sensitive mapping combined with CRL quantification is good at picking the correct alignments. For this reason, in chira mode there are ≥10% more perfect reads in all samples.

There is a decreasing trend in perfect reads for CLAN-based results on reads of lengths 15–20 nt with inserts, whereas it is more stable for BWA-MEM–based results. As this trend can also be seen in naive mode, it is likely more of a flaw of the aligner than ChiRA processing. For BWA-MEM–based alignments we consider an arm to be unmapped if it has no alignment on the sense strand. For this reason, there are many reads in the partial_miss and both_miss categories for BWA-MEM–based results even though there might be wrong alignments on the anti-sense strand.

For reads with shorter arms, even with very sensitive alignment settings, both aligners struggled to map to correct locations. Hence, we suggest tweaking the alignment settings of the aligners to capture read segments of ≥15 nt long. Shorter alignments often tend to be from ambiguous or wrong locations and eventually lead to false-positive interactions.

### Inferring CRL significance from published data

For the analysis of all published datasets, we used BWA-MEM to map the reads to reference databases and enabled CRL creation. From the process of creating CRLs, it is noticeable that the loci of a CRL share a common reference sequence. In this section we show that CRLs are not just random groups but have high sequence identity and that genes associated with the loci of a CRL implicate common annotations and functions.

#### CRLs and sequence identity

To determine the extent of the similarity among the CRL member loci, we computed the sequence identities. Each locus within a CRL is unique and does not contain any duplicate regions from gene isoforms. While running the workflow we used the default value of 0.7 for the option –crl_share_threshold. With this option loci having ≥70% of reads in common are grouped into a CRL. First, for each CRL we computed all pairwise global alignments among the loci using the Biopython module pairwise2 [41] with default alignment parameters. With no gap or mismatch penalties in default parameters, we essentially counted the number of matching bases. We then calculated the average of pairwise sequence identities (APSI) per CRL and a final mean per sample overall CRLs normalized by the CRL size. Pairwise sequence identity is the ratio of the alignment score to the average sequence length of the sequences. As a baseline, for each CRL size, we randomly sampled loci and computed the APSIs.

**Figure 4:**
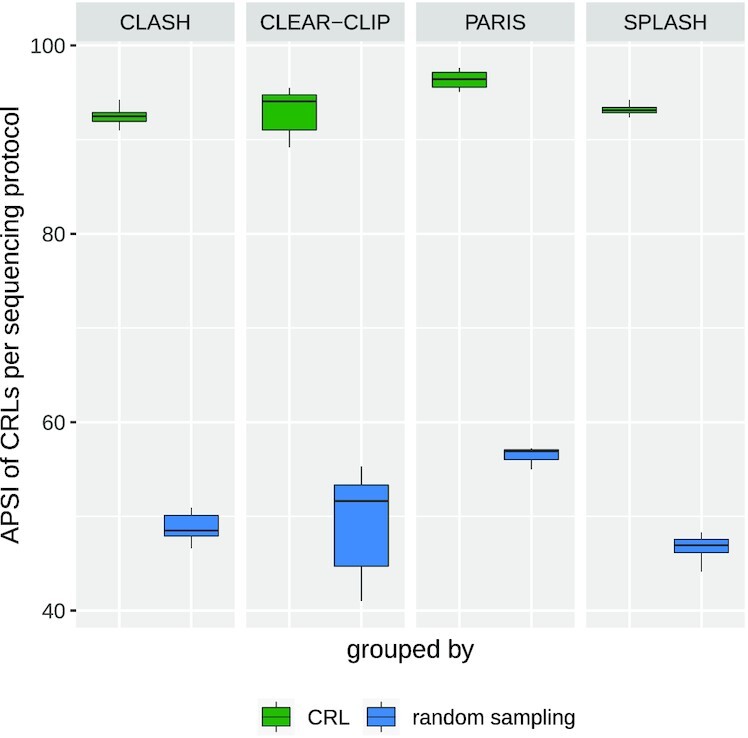
Box plots showing the average pairwise sequence identities (APSIs) among the member loci of the CRLs and those calculated from randomly sampled loci for each sequencing protocol. The loci sequences belonging to the CRLs show higher median of APSI than random sampling.

Fig. 4 shows the box plots of the APSIs over all the samples in each sequencing protocol. Notably, with a default value of 0.7 for CRL share, we see that all the protocols have a median of ≥90% APSI s, whereas the APSIs for randomly sampled loci are only ∼50%. This similarity among the CRL loci is compelling considering that the global alignment is used. It is also consistent across different sequencing protocols.

#### Biological relevance of CRLs

**Figure 5:**
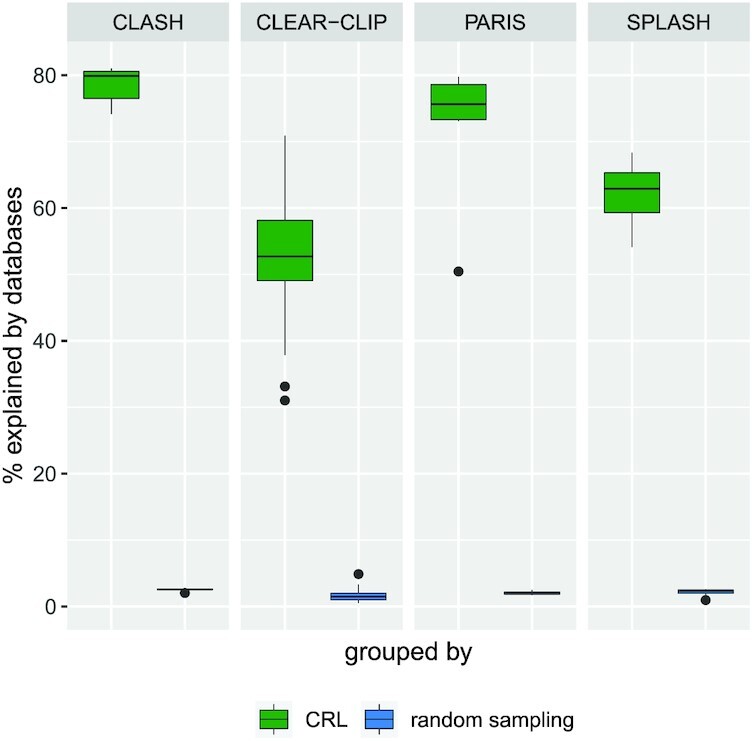
Validation of the CRLs from the different experimental protocols comparing the percentage of genes belonging to the CRLs that belong to the same protein family or have a similar KEGG identifier vs those when the genes were randomly sampled. In all datasets, it is clear that the genes constituting CRLs are found to be related in at least one of the annotation databases.

Robert and Watson [31] showed for a handful of genes that the groups of genes that are consistently multi-mapped are from gene families. Similarly, here on a large scale, we analyzed whether the genes that constitute the CRLs share biologically relevant information. We created an annotation database by extracting Rfam family, Ensembl protein family, and KEGG pathway information from Ensembl biomart [42]. We excluded all the CRLs from the analysis that do not contain ≥2 annotated genes in the database. For each CRL, we counted the number of genes with the same protein family or the same KEGG pathway or enzyme ID. We then calculated the ratio of this number to the total number of genes per CRL. In the end, we computed a weighted average over all the samples for each experimental protocol. As a control for each CRL, we randomly sampled the same number of genes out of the databases and calculated the percentage of those genes sharing a protein family or KEGG ID. Fig. 5 shows the box plots for the above explained values for CRL genes and randomly sampled genes for each experimental protocol. In all cases, it is evident that for most of the CRLs gene constitution is explainable compared to random gene constitution. Although not all of the CRLs have explainable sources (e.g., CLEAR-CLIP and SPLASH), overall the genes from a CRL more often belong to the same gene family or KEGG pathway than randomly sampled genes. Note that the CRLs are built from the short loci, which are just tiny portions of the genes. But here we are evaluating them at the level of the whole gene to which they belong. Although the loci are highly similar, the gene-level assessment might not necessarily explain all the CRLs.

### Sensitive chimeric read detection using ChiRA

**Figure 6:**
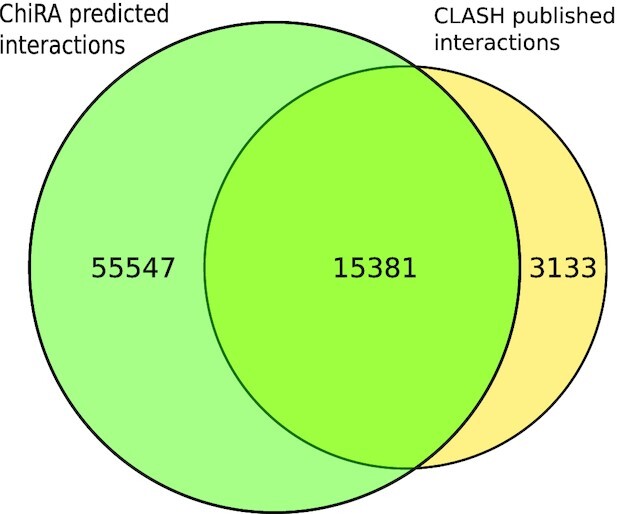
Number of interactions that were detected by ChiRA compared to published interactions in CLASH datasets.

**Figure 7:**
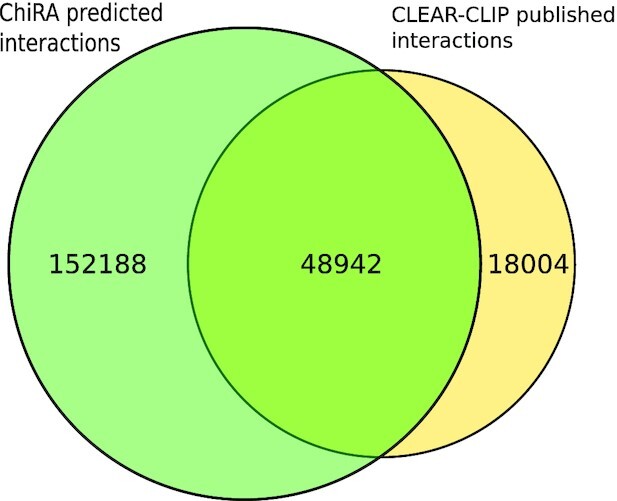
Number of interactions that were detected by ChiRA compared to published interactions in CLEAR-CLIP datasets.

Finally, we tested the sensitivity of ChiRA in detecting interactions by analyzing all CLASH and CLEAR-CLIP mouse datasets and subsequently comparing them with the published interactions. To be consistent with the published interactions, for CLASH we considered miRNA IDs with their target transcript positions and for CLEAR-CLIP miRNA IDs with their target genomic positions. Because we used the transcriptomic database for mapping, we ignored the intronic and intergenic target sites from CLEAR-CLIP published interactions. From ChiRA output, we selected chimeric reads with a final probability of ≥0.5 and the detected interacting loci that could be hybridized by IntaRNA. Figs. 6 and 7 show Venn diagrams intersecting the published interactions and interactions predicted by ChiRA for the CLASH and CLEAR-CLIP, datasets respectively. There is a large overlap of 83% with CLASH and 73% with CLEAR-CLIP published interactions despite using different aligners. Compared to the published dataset(s), ChiRA on average detects 3 times more interactions. Given our analysis of benchmark data (Figs. 2 and 3), and supported by IntaRNA hybridization of interacting loci, we assume that the majority of these detected interactions are true-positive results.

### Visualization of chimeric reads

**Figure 8:**
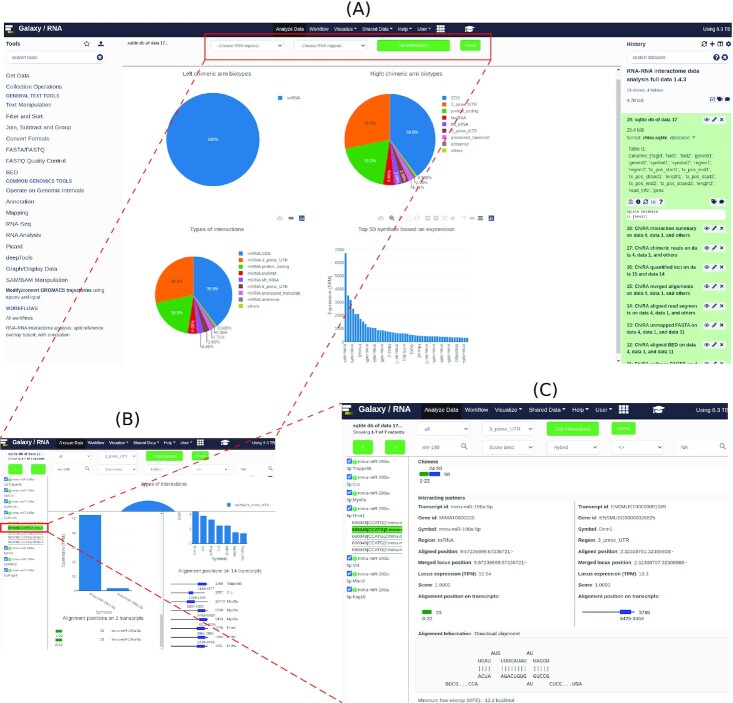
ChiRAViz Galaxy visualization. (A) The home page of the visualization. The plots on this page summarize the RNA biotypes of left and right chimeric arms, types of interactions, and highly abundant genes within the sample. (B) The second page shows the interactions of selected biotypes. On this page, users can further search, sort, and filter the interactions and obtain a deep summary of filtered interactions. (C) Interaction information page that shows such useful information as gene symbol, transcript IDs, gene IDs, expression level, biotypes, a depiction of interaction reference regions at transcript level, an illustration of the aligned read positions, and IntaRNA predicted hybrid.

The visualization has 3 views. The first page, shown in Fig. 8A, displays numerous plots to summarize the complete data. Two pie charts show the RNA biotype distribution of interacting transcripts. Another pie chart shows the distribution of interactions. Moreover, there is a bar plot that lists the gene symbols of top interacting transcripts sorted in decreasing order of their respective loci expressions. At the top of the page, there are 2 select boxes for choosing the interacting RNA types. When an interacting pair is chosen from these select boxes, it redirects to the second page (Fig. 8B), which shows all the interactions that involve these selected RNA biotypes. On the left, there is a list of unique combinations of gene symbols that represent unique RNA-RNA interactions. At the top of this page, there are several filters such as search and sort, which facilitate fetching data in the desired way. All or some of these entries can be selected together and a summary can be seen in the form of pie charts, histograms, and transcript-level alignment positions. The pie charts show distributions of the gene symbols and biotypes, and the histograms show the distributions of alignment scores and their loci expressions. The alignment regions on each interacting transcript are also depicted with the start, end, and length of the alignment. All the selected interactions can be exported as a tab-separated value file to the local computer. The pagination shown at the top left corner enables navigation through all the interactions and displays a small number of interactions (50) at a time, which simplifies the UI. All the unique reads associated with each interaction can be seen by clicking on the ”+” icon adjacent to the interactions themselves. Clicking on any of these single records displays the interaction summary page, as shown in Fig. 8C. This page shows all the information related to interacting partners such as gene ID, gene symbol, biotype, alignment start and end positions, transcript length, CIGAR string of the read alignment, and the expression of its corresponding locus in TPM. If there is an IntaRNA predicted hybrid, it is shown at the bottom of this page.

### Integration into Galaxy framework and tutorial

**Figure 9:**
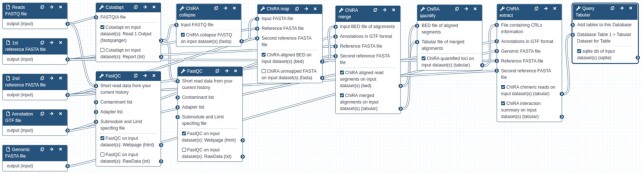
ChiRA Galaxy workflow. The workflow takes the FASTQ files that contain raw sequencing reads, process them, and produces a tabular and an SQLite database of interactions that are ready to be visualized by ChiRAViz.

Galaxy [25] has been one of the most popular resources for reproducible research. It allows easy execution of tools and complex workflows on a web-based graphical user interface. With public Galaxy servers, users also get access to huge computing resources. We integrated all of our tools intoGalaxy. The whole Python suite is available through Bioconda [43] and BioContainers [44] for easy installation. Galaxy Training Network (GTN) is a Galaxy community aimed at developing analysis-specific training material [45]. We developed training material for RNA-RNA interactome data analysis that includes a step-by-step guide to hands-on Galaxy analysis workflows with example datasets, ready-to-use Galaxy workflows, and an example Galaxy history. The training material also deals with the visualization framework. Being nicely coupled into the Galaxy ecosystem, ChiRA is now part of RNA workbench [46], a large comprehensive Galaxy-based web server for RNA-based research. All the data and ChiRA analysis discussed in this article are available through RNA workbench. Fig. 9 shows the ChiRA Galaxy workflow that uses split reference.

## Conclusion

In this article, we presented a comprehensive solution for RNA-RNA interactome and RNA structurome data analysis. Our method of creating CRLs from loci with consistent multi-mapped reads and quantification proved to rescue more reads from benchmark data. We also showed that the loci within a CRL have high sequence identities and the genes that constitute the CRLs originate from the same protein families or share common functional pathways, revealing that it is sensible to group consistently multi-mapped loci into CRLs. To our knowledge, ChiRA along with ChiRAViz is the only tool suite that makes analysis of RNA-interactome and structurome datasets easily accessible to users through Bioconda and Galaxy.

## Availability of Source Code and Requirements

- Project name: ChiRA
- Project home page: https://github.com/pavanvidem/chira
- Visualization: https://github.com/galaxyproject/galaxy/tree/dev/config/plugins/visualizations/chiraviz
- Operating system(s): Platform independent
- Programming language: Python
- Other requirements: Anaconda
- Installation: conda install -c conda-forge -c bioconda chira
- License: GNU General Public License Version 3
- Galaxy tool suite: https://github.com/galaxyproject/tools-iuc/tree/master/tools/chira
- Galaxy training tutorial: https://galaxyproject.github.io/training-material/topics/transcriptomics/tutorials/rna-interactome/tutorial.html
- Galaxy workflows:
https://rna.usegalaxy.eu/u/videmp/w/rna-rna-interactome-analysis (using BWA-MEM)
https://rna.usegalaxy.eu/u/videmp/w/rna-rna-interactome-analysis-using-clan (using CLAN)
- BiotoolsID: chira
- RRID:SCR_019219

## Data Availability

The benchmark data that were used to evaluate the performance of ChiRA can be obtained from Zenodo [47]. Snapshots of our code and other data are openly available in the GigaScience repository, GigaDB [48].

## Additional Files

Supplementary Section S2. Data and pre-processing.

Supplementary Section S3. Calculation of Transcripts per Million.

Supplementary Section S4. Data availability.

## Abbreviations

BAM: Binary sequence Alignment/Map; BED: Browser Extensible Data; BWA: Burrows-Wheeler Aligner; CIGAR: Compact Idiosyncratic Gapped Alignment Report; CLASH: Cross-linking Ligation and Sequencing of Hybrids; CLIP-seq: cross-linking immunoprecipitation sequencing; CRL: common read loci; EM: expectation-maximization; GTF: Gene Transfer Format; JS: JavaScript; KEGG: Kyoto Encylopedia of Genes and Genomes; LIGR-Seq: ligation of interacting RNA followed by high-throughput sequencing; miRNA: microRNA; ncRNA: non-coding RNA; nt: nucleotides; PARIS: Psoralen Analysis of RNA Interactions and Structures; PSI: pairwise sequence identities; SAM: Sequence Alignment/Map; SPLASH: Sequencing of Psoralen crosslinked, Ligated, and Selected Hybrids; UI: user interface; UTR: untranslated region.

## Competing Interests

The authors declare that they have no competing interests.

## Funding

This work was supported by the German Research Foundation (DFG) grant eCLASH: Definition des Interactomes kleiner RNA [2168/14-1 awarded to R.B.] and the DFG-funded Collaborative Research Centre 992 Medical Epigenetics [SFB 992/1 2012 awarded to R.B.]. The article processing charge was funded by the Baden-Württemberg Ministry of Science, Research and Art and the University of Freiburg in the funding programme Open Access Publishing.

## Authors' Contributions

P.V. implemented the ChiRA tool suite, integrated into Galaxy, created training material, analyzed the data, and wrote the major portion of the manuscript. A.K. developed the ChiRAViz Galaxy visualization and was involved in writing corresponding sections of the manuscript. B.A.G. and O.Z. supported in Galaxy integration and deployment. All authors were involved in reviewing the manuscript.

## Supplementary Material

giaa158_GIGA-D-20-00250_Original_Submission

giaa158_GIGA-D-20-00250_Revision_1

giaa158_GIGA-D-20-00250_Revision_2

giaa158_Response_to_Reviewer_Comments_Original_Submission

giaa158_Response_to_Reviewer_Comments_Revision_1

giaa158_Reviewer_1_Report_Original_Submission Anil Thanki -- 9/16/2020 Reviewed

giaa158_Reviewer_2_Report_Original_Submission Mallory Freeberg, Ph.D. -- 10/11/2020 Reviewed

giaa158_Supplemental_File

## References

[1] Ambros V . The functions of animal microRNAs. Nature. 2004;431(7006):350–5. 15372042 10.1038/nature02871

[2] Henras AK, Dez C, Henry Y. RNA structure and function in C/D and H/ACA s(no)RNPs. Curr Opin Struct Biol. 2004;14(3):335–43. 15193314 10.1016/j.sbi.2004.05.006

[3] Bartel DP . MicroRNAs: genomics, biogenesis, mechanism, and function. Cell. 2004;116(2):281–97. 14744438 10.1016/s0092-8674(04)00045-5

[4] Mattick JS, Makunin IV. Non-coding RNA. Hum Mol Genet. 2006;15(suppl_1):R17–R29. 16651366 10.1093/hmg/ddl046

[5] Plotnikova O, Skoblov M. Efficiency of the miRNA–mRNA interaction prediction programs. Mol Biol. 2018;52(3):467–77. 10.7868/S0026898418030187 29989587

[6] Helwak A, Kudla G, Dudnakova T, et al. Mapping the human miRNA interactome by CLASH reveals frequent noncanonical binding. Cell. 2013;153(3):654–65. 23622248 10.1016/j.cell.2013.03.043 PMC3650559

[7] Moore MJ, Scheel TKH, Luna JM, et al. miRNA–target chimeras reveal miRNA 3’-end pairing as a major determinant of Argonaute target specificity. Nat Commun. 2015;6:8864. 26602609 10.1038/ncomms9864 PMC4674787

[8] Lu Z, Zhang QC, Lee B, et al. RNA duplex map in living cells reveals higher-order transcriptome structure. Cell. 2016;165(5):1267–79. 27180905 10.1016/j.cell.2016.04.028 PMC5029792

[9] Aw JGA, Shen Y, Wilm A, et al. In vivo mapping of eukaryotic RNA interactomes reveals principles of higher-order organization and regulation. Mol Cell. 2016;62(4):603–17. 27184079 10.1016/j.molcel.2016.04.028

[10] Sharma E, Sterne-Weiler T, O’Hanlon D, et al. Global mapping of human RNA-RNA interactions. Mol Cell. 2016;62(4):618–26. 27184080 10.1016/j.molcel.2016.04.030

[11] Maher CA, Kumar-Sinha C, Cao X, et al. Transcriptome sequencing to detect gene fusions in cancer. Nature. 2009;458(7234):97–101. 19136943 10.1038/nature07638 PMC2725402

[12] Kannan K, Wang L, Wang J, et al. Recurrent chimeric RNAs enriched in human prostate cancer identified by deep sequencing. Proc Natl Acad Sci U S A. 2011;108(22):9172–7. 21571633 10.1073/pnas.1100489108 PMC3107329

[13] Asmann YW, Necela BM, Kalari KR, et al. Detection of redundant fusion transcripts as biomarkers or disease-specific therapeutic targets in breast cancer. Cancer Res. 2012;72(8):1921–28. 22496456 10.1158/0008-5472.CAN-11-3142

[14] Tandefelt DG, Boormans J, Hermans K, et al. ETS fusion genes in prostate cancer. Endocr Relat Cancer. 2014;21(3):R143–52. 24659477 10.1530/ERC-13-0390

[15] Lewis BP, Burge CB, Bartel DP. Conserved seed pairing, often flanked by adenosines, indicates that thousands of human genes are microRNA targets. Cell. 2005;120(1):15–20. 15652477 10.1016/j.cell.2004.12.035

[16] Esteller M . Non-coding RNAs in human disease. Nat Rev Genet. 2011;12(12):861–74. 22094949 10.1038/nrg3074

[17] Mendell JT, Olson EN. MicroRNAs in stress signaling and human disease. Cell. 2012;148(6):1172–87. 22424228 10.1016/j.cell.2012.02.005 PMC3308137

[18] Coolen M, Bally-Cuif L. MicroRNAs in brain development and physiology. Curr Opin Neurobiol. 2009;19(5):461–70. 19846291 10.1016/j.conb.2009.09.006

[19] Pinzón N, Li B, Martinez L, et al. microRNA target prediction programs predict many false positives. Genome Res. 2017;27(2):234–45. 28148562 10.1101/gr.205146.116 PMC5287229

[20] Broughton JP, Pasquinelli AE. A tale of two sequences: microRNA-target chimeric reads. Genet Sel Evol. 2016;48(1):31. 27044644 10.1186/s12711-016-0209-x PMC4819279

[21] Langmead B, Salzberg SL. Fast gapped-read alignment with Bowtie 2. Nat Methods. 2012;9(4):357. 22388286 10.1038/nmeth.1923 PMC3322381

[22] Li H . Aligning sequence reads, clone sequences and assembly contigs with BWA-MEM. arXiv 2013:1303.3997.

[23] Dobin A, Davis CA, Schlesinger F, et al. STAR: ultrafast universal RNA-seq aligner. Bioinformatics. 2013;29(1):15–21. 23104886 10.1093/bioinformatics/bts635 PMC3530905

[24] Travis AJ, Moody J, Helwak A, et al. Hyb: a bioinformatics pipeline for the analysis of CLASH (crosslinking, ligation and sequencing of hybrids) data. Methods. 2014;65(3):263–73. 24211736 10.1016/j.ymeth.2013.10.015 PMC3969109

[25] Afgan E, Baker D, Batut B, et al. The Galaxy platform for accessible, reproducible and collaborative biomedical analyses: 2018 update. Nucleic Acids Res. 2018;46(W1):W537–44. 29790989 10.1093/nar/gky379 PMC6030816

[26] Martin M . Cutadapt removes adapter sequences from high-throughput sequencing reads. EMBnet J. 2011;17(1):10–2.

[27] Zhong C, Zhang S. Accurate and efficient mapping of the cross-linked microRNA-mRNA duplex reads. iScience. 2019;18:11–9. 31271968 10.1016/j.isci.2019.05.038 PMC6609836

[28] Li H, Handsaker B, Wysoker A, et al. The Sequence Alignment/Map format and SAMtools. Bioinformatics. 2009;25(16):2078–9. 19505943 10.1093/bioinformatics/btp352 PMC2723002

[29] Langenberger D, Bermudez-Santana C, Hertel J, et al. Evidence for human microRNA-offset RNAs in small RNA sequencing data. Bioinformatics. 2009;25(18):2298–301. 19584066 10.1093/bioinformatics/btp419

[30] Holmqvist E, Wright PR, Li L, et al. Global RNA recognition patterns of post-transcriptional regulators Hfq and CsrA revealed by UV crosslinking in vivo. EMBO J. 2016;35(9):991–1011. 27044921 10.15252/embj.201593360 PMC5207318

[31] Robert C, Watson M. Errors in RNA-Seq quantification affect genes of relevance to human disease. Genome Biol. 2015;16(1):177. 26335491 10.1186/s13059-015-0734-x PMC4558956

[32] Zhang Z, Xing Y. CLIP-seq analysis of multi-mapped reads discovers novel functional RNA regulatory sites in the human transcriptome. Nucleic Acids Res. 2017;45(16):9260–71. 28934506 10.1093/nar/gkx646 PMC5766199

[33] Van Nostrand EL, Pratt GA, Yee BA, et al. Principles of RNA processing from analysis of enhanced CLIP maps for 150 RNA binding proteins. Genome Biol. 2020;21(1):90. 32252787 10.1186/s13059-020-01982-9 PMC7137325

[34] Teng M, Love MI, Davis CA, et al. A benchmark for RNA-seq quantification pipelines. Genome Biol. 2016;17(1):74. 27107712 10.1186/s13059-016-0940-1 PMC4842274

[35] Pachter L . Models for transcript quantification from RNA-Seq. arXiv 2011:1104.3889.

[36] Jin H, Wan YW, Liu Z. Comprehensive evaluation of RNA-seq quantification methods for linearity. BMC Bioinformatics. 2017;18(4):117. 28361706 10.1186/s12859-017-1526-y PMC5374695

[37] Xing Y, Yu T, Wu YN, et al. An expectation-maximization algorithm for probabilistic reconstructions of full-length isoforms from splice graphs. Nucleic Acids Res. 2006;34(10):3150–60. 16757580 10.1093/nar/gkl396 PMC1475746

[38] Mann M, Wright PR, Backofen R. IntaRNA 2.0: enhanced and customizable prediction of RNA–RNA interactions. Nucleic Acids Res. 2017;45(W1):W435–9. 28472523 10.1093/nar/gkx279 PMC5570192

[39] Griffiths-Jones S . miRBase: the microRNA sequence database. In: MicroRNA Protocols. Springer; 2006:129–38. 10.1385/1-59745-123-1:129 16957372

[40] Agarwal V, Bell GW, Nam JW, et al. Predicting effective microRNA target sites in mammalian mRNAs. Elife. 2015;4:e05005. 26267216 10.7554/eLife.05005 PMC4532895

[41] Cock PJ, Antao T, Chang JT, et al. Biopython: freely available Python tools for computational molecular biology and bioinformatics. Bioinformatics. 2009;25(11):1422–3. 19304878 10.1093/bioinformatics/btp163 PMC2682512

[42] Kinsella RJ, Kähäri A, Haider S, et al. Ensembl BioMarts: a hub for data retrieval across taxonomic space. Database (Oxford). 2011;2011, doi:10.1093/database/bar030. PMC3170168 21785142

[43] Grüning B, Dale R, Sjödin A, et al. Bioconda: sustainable and comprehensive software distribution for the life sciences. Nat Methods. 2018;15(7):475–6. 29967506 10.1038/s41592-018-0046-7 PMC11070151

[44] da Veiga Leprevost F, Grüning BA, Alves Aflitos S, et al. BioContainers: an open-source and community-driven framework for software standardization. Bioinformatics. 2017;33(16):2580–2. 28379341 10.1093/bioinformatics/btx192 PMC5870671

[45] Batut B, Hiltemann S, Bagnacani A, et al. Community-driven data analysis training for biology. Cell Syst. 2018;6(6):752–8. 29953864 10.1016/j.cels.2018.05.012 PMC6296361

[46] Fallmann J, Videm P, Bagnacani A, et al. The RNA workbench 2.0: next generation RNA data analysis. Nucleic Acids Res. 2019;47(W1):W511–5. 31073612 10.1093/nar/gkz353 PMC6602469

[47] Videm P . Benchmark data used in the evaluation of ChiRA tool-suite. Zenodo 2020. 10.5281/zenodo.4289365.

[48] Videm P, Kumar A, Zharkov O, et al. Supporting data for “ChiRA: an integrated framework for chimeric read analysis from RNA-RNA interactome and RNA structurome data.”. GigaScience Database; 2020. 10.5524/100845. PMC7844879 33511995

