# Supplementary material for: ChiRA: an integrated framework for chimeric read analysis from RNA-RNA interactome and RNA structurome data
Source: Gigascience. 2021 Jan 29;10(2):giaa158. doi: 10.1093/gigascience/giaa158 (PMC7844879; doi:10.1093/gigascience/giaa158)
Supplement: giaa158_GIGA-D-20-00250_Revision_1 [file giaa158_giga-d-20-00250_revision_1.pdf]

GigaScience

ChiRA: an integrated framework for Chimeric Read Analysis from RNA-RNA interactome and RNA structurome data

--Manuscript Draft--

Manuscript Number:	GIGA-D-20-00250R1
Full Title:	ChiRA: an integrated framework for Chimeric Read Analysis from RNA-RNA interactome and RNA structurome data
Article Type:	Technical Note
Funding Information:	Deutsche Forschungsgemeinschaft (2168/14) — Prof. Dr. Rolf Backofen<br>Deutsche Forschungsgemeinschaft (SFB 992/1 2012) — Prof. Dr. Rolf Backofen
Abstract:	Background: With the advances in next-generation sequencing technologies, it is possible to determine RNA-RNA interaction and RNA structure predictions on a genome-wide level. The reads from these experiments usually are chimeric with each arm generated from one of the interaction partners. Due to short read lengths, often these sequenced arms ambiguously map to multiple locations. Thus, inferring the origin of these can be quite complicated. Here we present ChiRA, a generic framework for sensitive annotation of these chimeric reads, which in turn to predict the sequenced hybrids.<br>Results: Grouping reference loci based on aligned common reads and quantification improved the handling of the multi-mapped reads in contrast to common strategies like the selection of the longest hit or a random choice among all hits. On benchmark data ChiRA improved the number of correct alignments to the reference up to 3-fold. It is shown that the genes that belong to the common read loci share the same protein families or similar pathways. In published data, ChiRA could detect 3 times more new interactions compared to existing approaches. In addition, ChiRAViz can be used to visualize and filter large chimeric datasets intuitively.
Corresponding Author:	Pavankumar Videm<br>Albert-Ludwigs-Universitat Freiburg Technische Fakultat<br>Freiburg im Breisgau, Baden-Württemberg GERMANY
Corresponding Author Secondary Information:	
Corresponding Author's Institution:	Albert-Ludwigs-Universitat Freiburg Technische Fakultat
Corresponding Author's Secondary Institution:	
First Author:	Pavankumar Videm
First Author Secondary Information:	
Order of Authors:	Pavankumar Videm
	Anup Kumar
	Oleg Zharkov
	Björn Andreas Grüning
	Rolf Backofen
Order of Authors Secondary Information:	
Response to Reviewers:	Dear Nicole Nogoy,<br><br>First of all, we would like to thank you for considering our manuscript for the GigaScience journal. We registered our tool-suite at bio.tools and scicrunch.org. Their corresponding IDs can now be found under the "Availability of Source Code and Requirements" section. We appreciate the reviewers for their valuable suggestions and constructive comments. With careful consideration to each of the reviewers' comments,

Powered by Editorial Manager® and ProduXion Manager® from Aries Systems Corporation

we updated the manuscript when needed. The changes in the manuscript are highlighted in red color. Please find a point-by-point response to the reviewers' comments below.
I am looking forward to hearing from you.
Sincerely yours,
Pavankumar Videm

Point-by-point response to the reviewer comments

REVIEWER 1:
Authors of the paper have put together an excellent workflow for RNA-interactome data analysis. Making the workflow available in Galaxy shows a great effort by authors to make it available for a wider audience. ChiRAViz is also a great addition to the work to visualise the results within Galaxy.
I am not an expert to judge the biological aspects of work, however, authors have tried their best to explain every aspect of the workflow in detail. I am reviewing the tool/software and visualisation aspects of the tool and manuscript.
Here, authors have put in a lot of effort to make their work available to a wider audience by making source code available from GitHub and installation from Conda package management system as well the whole workflow and intermediary tools as available in Galaxy public instance.
Authors also prepared Galaxy training material with test data and step by step guide to execute the workflow.
Following minor changes to manuscript and workflow will be good:

1. Authors have not provided a direct link to the workflow configuration file (.ga) for users to download and execute in local Galaxy, which will also allow tailoring workflow by swapping tools if needed. Though I managed to find workflow source code from Galaxy training material.

Authors' response: We updated the links to the workflows in the section "Availability of source code, workflow, and training material". Now they can be saved onto a local computer or imported into other Galaxy user accounts.

2. When running ChiRA, as a Galaxy workflow aligner option in ChiRAmap can only be changed in workflow editor view, it may be worthwhile to look into an option which allows changing aligner in workflow run view.

Authors' response: The aligner is implemented as a conditional parameter in Galaxy. This type of parameter can not be changed during runtime as it could change inputs as well. This is the current limitation of the Framework and discussed in this issue: https://github.com/galaxyproject/galaxy/issues/4528 To make it more convenient for the users, we created 2 separate workflows using BWA-MEM and CLAN.

3. I have run the workflow a couple of times and the output format for SQLite database is set to .sqlite, it needs to be converted into chira.sqlite to enable visualisation plugin. The format should have been set to chira.sqlite.

Authors' response: It is definitely a good idea to produce chira.sqlite so that it is readily usable by the visualization. The workflow now produces chira.sqlite by default.

4. Also, a Galaxy workflow overview image in the manuscript would be a good addition.

Authors' response: Thank you for the suggestion. We added a figure (Figure 9) of the Galaxy workflow and cited it in the section "Integration into Galaxy framework and tutorial".

5. In manuscript, section 'Visualization of chimeric reads' explains the visualisation well, but intext reference to figure and legend is missing which will help the reader to navigate while reading the text. The resulting SQLite database has one large table, I

would suggest splitting the database into separate tables to make it more scalable and also potentially reusable by other tools.

Authors' response: Thank you for your suggestion. We added references to each sub-figure based on the visualization page that is being explained. We also adapted the text to follow the steps in Figure 8. It is not necessary to split the table (horizontally) into multiple smaller tables as we provide table filtering and export features in the visualization (ChiraViz), which enables researchers and users to filter out smaller tables based on multiple parameters such as RNA biotypes, score, free energy, etc. These smaller tables can be exported/downloaded in a local machine and can be used as input files for different tools. Splitting the table vertically, which is storing related columns in different tables, would require joining all these tables (using join statements) during query time and also storing few identifier columns as foreign keys. This approach will significantly increase the query time. Therefore, we do not split the table into multiple tables.

REVIEWER 2:
The manuscript entitled ChiRA: an integrated framework for Chimeric Read Analysis from RNA-RNA interactome and RNA structurome data describes an approach for identifying RNA-RNA interactions from sequencing experiments that produce chimeric reads. The authors describe an alignment and quantification approach, including a new method to group reference loci to improve performance. The authors evaluate their approach using real published datasets and benchmark datasets. A notable strength of the manuscript is the availability of the ChiRA tools, a tutorial, and a workflow as part of Galaxy. These resources allow someone to easily reproduce the analyses presented in the manuscript or implement the ChiRA method for their own data. Overall the manuscript addresses a unique challenge, and the resources presented would be useful for anyone who generates or analyzes chimeric sequencing datasets.
I recommend this manuscript for publication after minor revisions, which are described below.

Introduction

1. The authors state that " To support computational methods, several transcriptome-wide experimental protocols have been developed recently to detect both inter- and intra-molecular RNA structure [6, 7, 8, 9, 10]. Although, these protocols vary in their application-specific details, they currently all involve ligating the two RNA interaction partners together and subsequently sequencing the resulting chimeric RNA molecules using high-throughput-sequencing technology. " (page 1). To be more comprehensive about approaches for detecting RNA structure, I encourage the authors to reference other types of protocols, e.g. approaches that use structure-specific enzymes to mark single- and double-stranded RNAs (e.g. PARS, DOI: 10.1038/nature09322).

Authors' response: Although the reviewers' suggestion to include PARS like protocols make it comprehensive, we did not add them for the following reason. PARS data could only be integrated to improve the accuracy of the structure prediction as it has no information about base-pair complementarity. However, it is rarely the case to have PARS and structurome data (like SPLASH or PARIS) from the same experiment. Thus, we believe that it is at best a minor improvement in our setting but may also introduce noise. Therefore, we assume that referring these methods may bring more confusion to the readers.

2. It would strengthen the motivation of the work and provide broader interest if the authors mentioned how their approach could be applied in contexts other than identifying miRNA:mRNA interactions, e.g. to identify chimeric RNAs in cancer or structural genome rearrangements, to name a few examples.

Authors' response: We included a short text regarding this at the end of the first paragraph of the introduction.

Powered by Editorial Manager® and ProduXion Manager® from Aries Systems Corporation

3. The Introduction ends a bit abruptly (page 2). I suggest adding a few sentences at the end that summarise how this work addresses the challenges described at the end of the Introduction, and highlight how the work can contribute broadly to scientific research.

Authors' response: We added a few sentences at the end of the introduction to summarize the contribution of our method. We also moved some text from the second paragraph ("Existing software solutions ... identified RNA-RNA interactions) to the end of the section where it best fits best.

Methods

1. Unique molecular identifiers are mentioned (page 2), but UMIs are still relatively new. I suggest the authors provide a little more background information on UMIs, specifically to help clarify to readers how deduplication by UMI is distinct from standard deduplication, which is an important point the authors make in this section.

Authors' response: We added a couple of sentences to explain the UMIs, the reason behind using them, and how we deduplicate based on UMIs.

2. The colored reads in Figure 1 ("Reads (FASTQ)", "whole/split reference (FASTA)") are confusing. Read deduplication is represented (removal of a dark blue read), but the brown read disappears and then reappears in the alignment step, and there are colored reads in the alignment step that don't appear in the first step. I suggest ensuring that the representation of the method in Figure 1 is consistent with the described method.

Authors' response: We thank the reviewer for this important observation. We changed the read colors in Figure 1 and made sure that these colors are consistent throughout the figure. Additionally, we changed the representation of the "Building common read loci" and "Quantification of CRLs" steps. Now the quantification is shown as bars that are generated by the read segment counts for each CRL. We also extended the figure caption with more explanation on that particular example in the figure.

3. The section on choosing which reference to align to (page 4) would benefit from more discussion on the pros and cons of choosing the genome vs the transcriptome. The authors provide two reasons for choosing to align to the transcriptome, but it would be interesting to hear the authors' thoughts on whether aligning to the transcriptome could be affected by how well-defined an organism's transcriptome is. Especially in the context of miRNA:target mapping, where many miRNA targets are in 3'UTRs, organisms with poorly annotated transcriptomes (missing isoforms where expression is cell- or tissue- or developmental-specific) or poorly defined 3'UTRomes will potentially miss mapping (higher false negatives). These mappings could be recovered if reads are aligned to the genome. I think readers would benefit from an expansion of this section.

Authors' response: We agree that giving reasons to align to transcriptome only seems to be incomplete. We extended the text and gave reasons to map to the whole genome, as suggested by the reviewer.

Results and Discussion

1. "Based on the benchmark data provided by the CLAN publication, we produced our benchmark data to test the performance of ChiRA." (page 6). It would be great if the authors could publish or make available the benchmark data they generated and used, for example in the ChiRA GitHub repository.

Authors' response: We created a Zenodo dataset with the benchmark data (https://zenodo.org/record/4289365) and added a citation from the section "Availability of supporting data and materials".

2. "Each read is a direct fusion of (sub)sequences of human hg38 miR-Base [34] mature miRNAs and a random TargetScan [35] target sequence (i.e., the target sequence is not necessarily a true target of this miRNA)." (page 6). If the benchmark

data is made of TargetScan predicted target sequences, does this bias the benchmark data for computationally predicted interactions, which has potentially many false positives?

Authors' response: We believe that there is no bias in this regard, as we did not optimize any parameters or built any models to guide our method. Please note that there is no relation between the fused sequences. We used benchmark data to show that our method can pick the correct alignments out of all possible multi mappings. Note that unlike CLAN benchmark data, our benchmark data contain reference sequences of varying lengths. Hence, we believe that it is a robust evaluation.

3. In Figure 4, the observation that PSI > 90% for all methods supports the authors' conclusion. The experiment would be stronger if the authors included a baseline PSI, for example using generated random regions of the reference or some form of random sampling, like was done for Figure 5.

Authors' response: Thank you for the suggestion. We did a random sampling of sequences and computed PSI as a baseline for Figure 4 and updated the text.

4. "Though not all of the CRLs have explainable sources (for eg, CLEAR-CLIP and SPLASH), they are far better than randomly sampled genes." (page 8). I agree with the conclusion presented, but I suggest using objective language instead of the subjective "far better" to describe that genes from the same CRL are more often found in the same gene family or KEGG pathway than randomly selected genes.

Authors' response: We take the suggestion from the reviewer and changed the sentence to "Although not all of the CRLs have explainable sources (for eg, CLEAR-CLIP and SPLASH), overall the genes from a CRL are more often belong to a same gene family or KEGG pathway than randomly sampled genes".

5. "There is a large overlap of 83% with CLASH and 73% with CLEAR-CLIP published interactions despite using different aligners. Compared to the published dataset(s), ChiRA on average detects three times more interactions." (page 9). I am wondering what the authors conclude from this result. Do they think ChiRA is producing many more false positives? Identifying more true positives than CLASH or CLEAR-CLIP?

Authors' response: Thank you for your question. Unfortunately, there is no "ground truth" dataset to our knowledge available to test whether the detected interactions are true positives or not. From our analysis of benchmark data and supported by IntaRNA hybridization of interacting loci, it is likely that the majority of these detected interactions are true positives. We added a sentence at the end of the section "Sensitive chimeric read detection using ChiRA".

Other

1. The Galaxy tutorial was informative and straightforward to follow. I greatly appreciate the authors making this available, and I think readers will find it very helpful. When I get to the "Viewing individual interaction information" section, step "Click on one of the records to view following information", nothing shows up in the middle panel when I select one of the records (using Galaxy Europe). I don't propose this is required for publication, but I suggest QA testing to ensure this visualisation works for users.

Authors' response: To view individual interaction information, the "+" icon should be clicked which shows all the records having the same combination of gene symbols on the left panel. Clicking on one of these records will show relevant information about the selected interaction in the middle panel. Alternatively, one or more checkboxes can be selected and then clicking on the summary button will show a summary of all the selected interactions in the middle panel. We updated the training material text and the figure with highlighted details on how to view a single record.

2. Competing Interests and Authors' information sections appear to contain boilerplate text. Either remove these sections or replace with relevant text.

Authors' response: Thank you for pointing this out. We modified the Competing

	Interests section and removed the Authors' information section.
Additional Information:	
Question	Response
Are you submitting this manuscript to a special series or article collection?	No
**Experimental design and statistics**<br><br>Full details of the experimental design and statistical methods used should be given in the Methods section, as detailed in our Minimum Standards Reporting Checklist. Information essential to interpreting the data presented should be made available in the figure legends.<br><br>Have you included all the information requested in your manuscript?	Yes
**Resources**<br><br>A description of all resources used, including antibodies, cell lines, animals and software tools, with enough information to allow them to be uniquely identified, should be included in the Methods section. Authors are strongly encouraged to cite Research Resource Identifiers (RRIDs) for antibodies, model organisms and tools, where possible.<br><br>Have you included the information requested as detailed in our Minimum Standards Reporting Checklist?	Yes
**Availability of data and materials**<br><br>All datasets and code on which the conclusions of the paper rely must be either included in your submission or deposited in publicly available repositories (where available and ethically appropriate), referencing such data using a unique identifier in the references and in the "Availability of Data and Materials" section of your manuscript.	Yes

Have you have met the above requirement as detailed in our Minimum Standards Reporting Checklist?	

Manuscript

Click here to access/download;Manuscript;GIGA-D-20-00250_revised_manuscript.pdf

GigaScience, 2020, 1–13

doi: xx.xxxx/xxxx
Manuscript in Preparation
Paper

PAPER

ChiRA: an integrated framework for Chimeric Read Analysis from RNA-RNA interactome and RNA structurome data

Pavankumar Videm[1], Anup Kumar[1], Oleg Zharkov[1], Björn Andreas Grüning[1] and Rolf Backofen[1,2*]

[1]Bioinformatics Group, Department of Computer Science, University of Freiburg, Georges-Koehler-Allee 106, 79110 Freiburg, Germany and [2]Signalling Research Centres BIOSS and CIBSS, University of Freiburg, Schaenzlestr. 18, 79104 Freiburg, Germany

*backofen@informatik.uni-freiburg.de

Abstract

Background: With the advances in next-generation sequencing technologies, it is possible to determine RNA-RNA interaction and RNA structure predictions on a genome-wide level. The reads from these experiments usually are chimeric with each arm generated from one of the interaction partners. Due to short read lengths, often these sequenced arms ambiguously map to multiple locations. Thus, inferring the origin of these can be quite complicated. Here we present `ChiRA`, a generic framework for sensitive annotation of these chimeric reads, which in turn to predict the sequenced hybrids.
Results: Grouping reference loci based on aligned common reads and quantification improved the handling of the multi-mapped reads in contrast to common strategies like the selection of the longest hit or a random choice among all hits. On benchmark data `ChiRA` improved the number of correct alignments to the reference up to 3-fold. It is shown that the genes that belong to the common read loci share the same protein families or similar pathways. In published data, `ChiRA` could detect 3 times more new interactions compared to existing approaches. In addition, `ChiRAViz` can be used to visualize and filter large chimeric datasets intuitively.

Key words: miRNA; chimeric read; RNA-RNA interactome; structurome; visualization; CLASH; CLEAR-CLIP; PARIS; SPLASH; Galaxy workflow

Introduction

Many non-coding RNAs (ncRNAs) regulate gene expression, post-transcriptionally, via mechanisms such as activation or inhibition of translation, destabilization, localization, and processing. For example, a microRNA can down-regulate target expression via translational inhibition or transcript destabilization, initiated by the formation of base pairs between the mature microRNA (~22 nt long) and the target RNA transcript [1]. For successful regulation, not only the inter-molecular structure (i.e., the RNA-RNA interaction) but also the structure of the ncRNA itself (i.e., the intra-molecular RNA structure) is key to the regulatory process [2, 3, 4], as it influences the parts of the ncRNA that are accessible for RNA-RNA interactions. Computationally, the prediction of both inter- and intra-molecular structure is non-trivial and results can be unreliable [5]. To support computational methods, several transcriptome-wide experimental protocols have been developed recently to detect both inter- and intra-molecular RNA structure [6, 7, 8, 9, 10]. Although these protocols vary in their application-specific details, they currently all involve ligating the two RNA interaction partners together and subsequently

Compiled on: November 26, 2020.
Draft manuscript prepared by the author.

Key Points

- ChiRA tool suite provides a complete analysis and visualization framework along with ready-to-use Galaxy workflows and tutorials for RNA-RNA interactome and structurome datasets
- Common read loci built by ChiRA can rescue multi-mapped reads on paralogous genes without requiring any information on gene relations.
- ChiRA is sensitive in detecting new RNA-RNA interactions from published RNA-RNA interactome datasets

sequencing the resulting chimeric RNA molecules using high-throughput-sequencing technology. Chimeric RNAs from gene fusions by trans-splicing or chromosomal rearrangements can be seen also in RNA sequencing data. Such chimeric RNAs are often associate with specific cancer types [11, 12] and considered to be potential biomarkers [13, 14].

MicroRNAs have been a subject of avid research in the last decade due mostly to two reasons: (1) it is proposed that each microRNA can regulate up to several hundred targets and that a substantial proportion of protein-coding genes are targeted by microRNAs at some stage [15] and (2) individual microRNAs have been implicated in several notorious human diseases, such as different cancer types and neuro-degenerative illnesses [16, 17, 18]. Therefore, accurate identification of microRNA targets is highly sought after. Despite numerous attempts, computational prediction approaches still deliver poor results with generally high false-positive rates with no significant improvement observed in the past decade (see review [19]). Therefore, considerable effort has also gone into developing high-throughput experimental protocols, specifically designed to detect miRNA-target interactions (reviewed in [20]). The most recent line of development has been to ligate the microRNA to the site-specific interaction region of the target, selecting these interactions via cross-linking to one of the Argonaute proteins required for microRNA-based regulation, and to sequence the resulting chimeric RNA molecule, for example, CLASH [6] and CLEAR-CLIP protocols [7]. Going beyond microRNAs, these protocols can obviously be applied to RNA interactions that involve a regulatory protein other than Argonaute. To generalize even further, researchers have applied the same idea to the detection of all transcriptome-wide RNA-RNA interactions. This includes both inter- and intra-molecular base-pairing without the necessity of choosing a specific regulatory protein for cross-linking, as done for example in PARIS [8], SPLASH [9], and LIGR-Seq [10]. Regardless of the protocol, the sequenced reads are chimeric, i.e., a fusion of either two different RNA fragments corresponds to inter-molecular interaction or two distinct parts of a single RNA molecule from its intra-molecular structure.

Two main computational challenges arise from such chimeric-read data: (1) mapping the chimeric reads to two different locations on reference transcript annotations and (2) dealing with the fact that these *short* RNA segments map to multiple locations, i.e., specifically dealing with multi-mapped reads. State-of-the-art mapping software, such as Bowtie2 [21], BWA-MEM [22], and STAR [23], can both map chimeric reads and allow for multiple mapping locations, given the appropriate parameter settings. Subsequent to mapping, however, there are no satisfactory or standard solutions for correctly quantifying multi-mapped reads. Multi-mapped reads are either ignored, incorrectly assigned and/or quantified. Three common approaches exist for assigning multi-mapped reads: (1) They are not assigned but simply discarded; (2) a read is assigned to each of the multi-mapped locations with equal distribution (e.g. with a count of one divided by the number of locations); and (3) the true expression level is estimated by assigning the read to a multi-mapped location proportionally to the number of uniquely mapped reads in the vicinity of that location. The ability to result read counts to capture expression levels or RNA-interaction events increases with each approach. Obviously, discarding multi-mapped reads is a poor solution and definitely not an option when dealing with chimeric reads. Distributing counts equally under- or overestimates the actual expression in all locations in comparison to regions with uniquely mapping reads. The third approach can deliver accurate results, however fails when it comes to distributing reads among gene families with very similar sequences, e.g. for microRNA gene families.

Existing software solutions that take the raw-data input from RNA-interactome protocols and deliver quality interaction annotations are currently application or protocol specific. Most of them were released along with their corresponding published experimental protocols and none of them has become a readily usable bioinformatic pipeline. There also exist generic standalone pipelines like Hyb [24], which was developed and demonstrated to deal with microRNA-specific data. From the computational side, there is thus still a major hurdle to overcome before such protocols can be broadly applied in practice: the availability of easy-to-use software that can process the raw data to produce accurate annotation and quantification of the identified RNA-RNA interactions. Here we present a method to resolve multi-mapping to very similar reference sequences from possible gene families and paralogs without requiring any prior annotation. Our method determines the best alignment for each multi-mapped read by an elegant quantification and scores them based on the abundance of reference loci. Our ChiRA tool suite, Galaxy [25] workflows and visualization provide a complete analysis framework for chimeric reads from RNA-RNA interactome and RNA structurome protocols. Thus, we aim to strengthen a weak link in the search for transcriptome-wide RNA interactions/structures.

Methods

We built a complete workflow that takes raw sequencing reads as input and outputs a comprehensive list of annotated interacting regions. This involves read deduplication, mapping, quantification (including multiple mapped reads) of reference loci to infer the correct locations based on their expression and hybridization of interacting reference loci. To offer a convenient interface on top of ChiRA output an interactive visualization ChiRAViz was developed. Figure 1 shows the complete workflow built from ChiRA and ChiRAViz tool suite. Each of the following sections corresponds to the steps represented (written on the right side) in the figure.

Adapter clipping and read deduplication

Quality and adapter trimming, in general, are crucial for RNA-RNA interactome data but essential for small RNA related interactome data. Mature miRNAs that interact with the targets

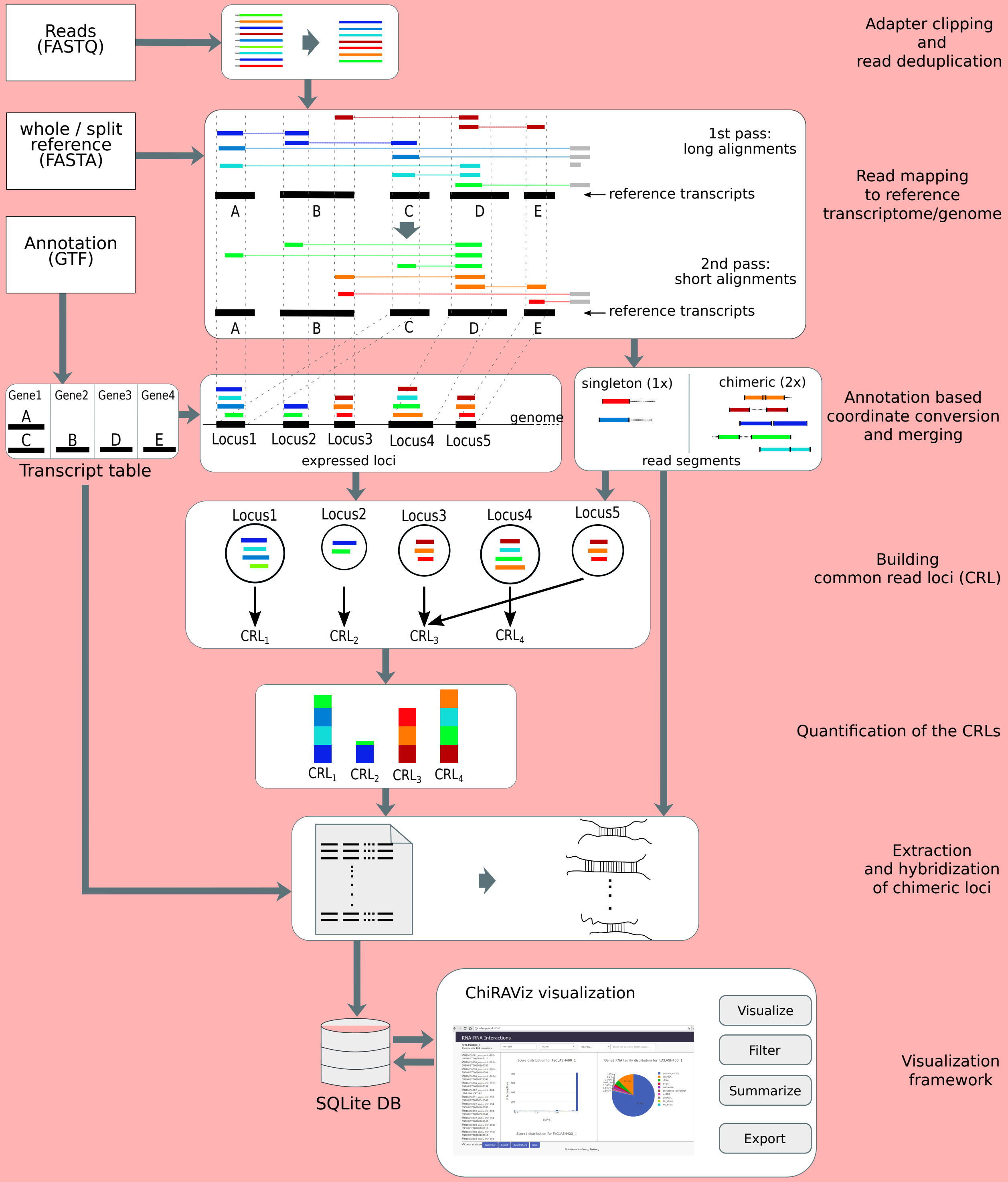

Figure 1. `ChiRA` workflow. First, the reads are deduplicated and mapped to reference sequences. Then the overlapping reference regions are merged into expressed loci. Given an annotation file, transcriptomic alignment positions are converted into genomic positions. Common read loci are built based on the reads that are consistently multi-mapped among the expressed loci. The quantification is carried out at the common read loci level, and the interactions are scored and hybridized. With the visualization, users can search, filter, and export preferred interactions. Here transcripts A and C are two isoforms of a gene *Gene1*. Because of shared exons, multi mappings to A and C can be collapsed into a single genomic locus *Locus1*. As *Locus3* and *Locus5* share all their read segments, they are merged into a single CRL. Due to the quantification based on Expectation-Maximization, the multi-mapped green read segment is counted more towards CRL_1 than CRL_2.

are only about 18-22 nt in length. Depending on the captured target sequence, chimeric reads often have adapters in them. In our analysis, at least 80% sequenced reads from CLASH and CLEAR-CLIP datasets contained adapters. For our analysis we trimmed low-quality ends and adapters from the reads using `cutadapt` [26]. Reads that are shorter than 10 nucleotides were discarded and the remaining reads are deduplicated to eliminate possible PCR duplicates. In general, not all identical reads are PCR duplicates. Gene isoforms or gene paralogs also result in duplicate RNA fragments. To uniquely identify the RNA fragments, unique molecular identifiers (UMI) are used. UMIs are short sequences of a specific length that are usually attached at the 5′ end of the RNA fragments during the library preparation. We also deduplicate reads based on UMIs if they present in the library. We consider identical reads with the same UMI as PCR duplicates, whereas identical reads with different UMIs as unique. The deduplication step may reduce the number of reads by orders of magnitude, which in turn can speed up the subsequent steps.

Read mapping to reference transcriptome/genome

In this step, we align the reads to the reference transcriptome or genome. For well-annotated organisms, we recommend using the transcriptome for the following reasons. i) When mapped against a transcriptome, reads can be mapped linearly across the splice junctions. Especially, in the case of these small read fragments, it can be extremely difficult to map across the splice junctions when mapped to the genome. ii) There is less chance of getting random false positive hits for short read fragments on transcriptome than on whole genome. Unfortunately, except for some model organisms, reference annotations are either incomplete or unreliable. In that case, using the whole genome sequence as a reference is a good choice for the following reasons. i) An unreliable annotation leads to false conclusions on the type of detected interactions. ii) An incomplete annotation results in false-negative interactions. Consider an example of CLASH data which predominantly contain miRNA and 3′ untranslated regions (UTR) interactions. Mapping to a reference transcriptome with an incomplete 3′ UTR annotation fails to capture the most important category of interactions.

Currently, we support mapping with `BWA-MEM` [22] and `CLAN` [27]. `CLAN` is a recent exclusive chimeric read mapper and outputs the chimeric alignments in tabular format. `BWA-MEM` is also capable of producing chimeric reads by local alignment. With a high dynamic range in read lengths, it is not always possible to accurately map chimeric reads of different lengths with a single parameter setting. Hence, when `BWA-MEM` used as the aligner, we do a two-pass alignment. The first pass targets mapping long chimeric read segments whereas the second pass at short ones. In the first pass, we use high alignment score thresholds and allow gaps and hence achieve long gapped chimeric alignments. In the second pass, we use a lower alignment score cut-off and do not allow any insertions or deletions. Therefore the second pass rescues short chimeric read segments with perfect matches on the reference. The default alignment settings were optimized on the miRNA interactome data from CLASH and CLEAR-CLIP protocols. The complete list of alignment settings can be found in the provided Galaxy histories (see supplementary section S4 for more details). `BWA-MEM` can output the alignments in Sequence Alignment/Map (SAM) format. We convert it into Binary sequence Alignment/Map (BAM) and use pysam [28] for further processing. It is important to consider that `BWA-MEM` randomly chooses one of the alignments as primary and writes all the alternative hits to the `XA` tag of the alignment. The true alignment can also be hidden under `XA` tag and buried in the BAM file. `BWA-MEM` has an option (*-h*) that controls the writing of these suboptimal alignments to the output BAM file. In the second pass, we set it to a high number (default 100) so that we do not miss any of the equally good alternative alignments. The idea is to get as many multi hits as possible and let `ChiRA` pick the best one in subsequent steps. In the end, we combine the alignments from both the alignment steps, parse the BAM file using pysam and write them to a Browser Extensible Data (BED) file. In this step, we only keep the alignments that are mapped on the sense reference strand. If there is an `XA` tag for an alignment we keep all the alternative alignments with the highest read coverage. In the end, we remove any duplicate hits due to two-pass alignment.

As each chimeric read often contains two RNA fragments originating from two different RNA types, we allow mapping to two different reference transcriptomes (*split reference*). For example, for CLASH data, we encourage to use a *split reference*. One containing miRNAs and the other containing the rest of the transcriptome which restricts the output to miRNA-based interactions. The parameters like seed lengths and alignment scores are dependent on the type of the data or expected length of chimeric arms. In our experience, the default settings work well with the miRNA interactome data.

Annotation-based coordinate conversion and merging

Given an annotation file in Gene Transfer Format(GTF), we convert transcriptome locations to genomic locations, as working on the genomic locations is less ambiguous. The main problem with transcript locations is that the reads mapped to the exons that are shared among the isoforms appear to be multi-mapped. But at the genomic level, these are uniquely mapped. In absence of GTF file `ChiRA` can still work with transcriptome locations.

Merge reference positions to define interaction sites

As the experimental protocols may generate several reads covering different parts of an interaction site, we have to define an interaction site by combining overlapping alignments. This step separates alignments stemming from the same interaction sites from alignments that cover a completely different interaction site on the same transcript. For example, two different miRNAs may target a single mRNA at two different locations like coding sequence and 3′ UTR. In more detail, we merge the significantly overlapping alignments based on the reference mapping locations to generate so-called *expressed loci*. A single transcript may have multiple such expressed loci. For an alignment to merge into an existing expressed locus, both the alignment and the locus must reciprocally overlap more than 70% (default value) in length.

While this approach works well with interaction sites that have a low to medium coverage, it might fail in the case of sites with high coverage, as the likelihood to find two alignments with 70% overlap at random increases. For this purpose, we have an alternative merging mechanism using `blockbuster` [29]. The `blockbuster` defines the blocks of alignments based on a Gaussian approximation of the read coverage. Subsequently based on `-distance` parameter, it places adjacent read blocks in to clusters. However, we ignore this cluster information and work further on the block level. We merge any overlapping blocks to define potential interaction loci. This approach is thus similar (but also simpler) to the one introduced and successfully applied for CLIP-seq peak calling in Holmqvist *et al.* [30].

Merge read positions to define chimeric arms

In this step, we identify all chimeric and non-chimeric (*singleton*) aligned reads. A chimeric read has at least two non-overlapping portions on the read mapped to distinct reference loci. If a sequenced read is chimeric and it is uniquely mapped to the reference, then we have at most 2 alignments each belonging to one chimeric arm. If a sequenced read is a singleton and mapped uniquely, then we have maximally one alignment. We call each aligned portion of the read as a *read segment*. In later steps, while quantification, a singleton read will be treated as one read whereas a chimeric read as two (one for each segment) separate reads. Hence it is crucial to define the chimeric split points of the reads. A chimeric split point can be identified by its non-overlapping segments. Due to local alignment and repetitive parts on the reference sequences, some overlapping segments multi-map with few bases shifted. Considering each such highly conserved read segment separately penalizes the overall read segment contribution in quantification. Hence, we further merge read segments that overlap at least 70% (default value) of their length into a single segment. In theory, there are only two interacting read segments as there are maximally two interacting RNA fragments captured in the interactome experiments. Due to sensitive alignment settings, some reads also result in more than two segments. After a subsequent quantification step, only the two most probable chimeric arms will be considered for each read.

Building common read loci

There are cases where read segments map to the gene families or paralogous loci sharing the common sequences. It is huge a challenge to find a decent annotation that carries gene family or paralogue information. It was shown in Robert & Watson *et al.* [31] that grouping of genes based on multi-mapped reads resulted in groups of gene families and analyzing the RNA-seq data at this group level was biologically relevant. Similarly, we propose a method to group multi-mapped loci which doesn't depend on any annotation. If two loci share a large portion of their multi-mapped reads, their sequences tend to be very similar or originate from the same gene families or paralogs or have similar pathways (see Results and Discussion). Hence, we group expressed loci into *common read loci* (CRL) if they share a significant number of multi-mapped reads. Here we use single-linkage clustering with the *Jaccard Index* to measure the similarity between the expressed loci. To merge an expressed locus into an existing CRL, the *Jaccard index* of sets of reads between that locus and the CRL should be greater than a user-defined threshold (default of 0.7). We merge the loci in order by size. If a locus failed to share a significant portion of multi-mapped reads with any other CRL, then it gets its own CRL. If the reads were mapped to transcriptome and the user does not provide any gene annotation file, CRLs are well capable of grouping multi-mapped reads that map to gene isoforms. See Algorithm 1 for CRL creation pseudo code.

Algorithm 1: CRL creation from expressed loci. $\mathbb{C}$ is the list of CRLs; $\mathbb{L}$ is the list of expressed loci; L_i is the set of read segments of an expressed locus i and C_k is the set of read segments of a CRL k.

Result: List of CRLs
$\mathbb{C} \leftarrow \{\}$;
for $L_i \in \mathbb{L}$ **do**
  `match` $\leftarrow$ *False*;
  for $C_k \in \mathbb{C}$ **do**
    if $\frac{C_k \cap L_i}{C_k \cup L_i} \geq \theta$ **then**
      $C_k \leftarrow C_k \cup L_i$;
      `match` $\leftarrow$ *True*
    end
  end
  if *not* `match` **then**
    $\bigcup\{\mathbb{C}, L_i\}$;
  end
end

Quantification of the CRLs

To score the mapped chimeric reads, we first need to estimate the expression of the CRLs by quantification. Quantification helps to assess the true origin of a read segment in the case of multi mapping. It has been shown that proper quantification of multi-mapped reads led to the discovery of novel protein-RNA interactions from CLIP-seq data [32, 33]. A study on RNA-seq data revealed that the expression of genes with multi-mapped reads was underestimated by common quantification methods [31]. There exist comprehensive studies on methods [34, 35] and metrics [36] for quantification of RNA-seq data but direct application of these methods to our data is not possible for the following reasons. First, it is hard to supply our pre-built locus-CRL relations to the quantification tools on the fly. Second, unlike our short reference loci, the reference RNAs in RNA-seq have multiple exons and are much longer. In RNA-seq, often the quantification is done at the isoform level, where exons that are unique to that isoform help to resolve the multi-mapping by estimating the total maximum likelihood for that isoform. But in interactome data, there is only a part of interacting exons captured and the rest is missing. If this interacting part of an exon is shared among the isoforms, the read segments mapped are still called multi-mapped and each transcript gets an equal share from the read segment. Therefore we implemented an approach to quantify the CRLs based on the Expectation-Maximization (EM) algorithm. In this quantification, all multi-mapped reads that map to different expressed loci of a CRL are considered as uniquely mapped to that CRL.

Let $\mathbb{S}$ be the set of all read segments with $N = |\mathbb{S}|$ and $\mathbb{C}$ be the set of all CRLs with $K = |\mathbb{C}|$. We follow Xing *et al.* [37] in the annotation, where we estimate the CRL abundance by determining the likelihood $\rho_c = Pr[s \in c]$ that a read segment s actually stemmed from CRL c. We denote with ρ the vector of all ρ_c. Note that when the CRLs have a similar length as in our case, length normalization can be omitted, i.e., ρ_c are then direct estimates for CRL abundances. In the case of multiple mapping, we define two indicator variable matrices to model the read segment selection process. We have an $N \times K$ indicator matrix $Z = (z_{s,c})_{\substack{s\in\mathbb{S}\\ c\in\mathbb{C}}}$ with

$$z_{s,c} = \begin{cases} 1 & \text{if read segment } s \text{ is from CRL } c \\ 0 & \text{else} \end{cases}$$

However, this is not directly observable in the case that the reads map to different CRLs. This can be overcome by introducing another matrix $Y = (y_{s,c})_{\substack{s\in\mathbb{S}\\ c\in\mathbb{C}}}$ with

$$y_{s,c} = \begin{cases} 1 & \text{if read segment } s \text{ maps to CRL } c \\ 0 & \text{else} \end{cases}$$

Note that we have in each row of Z exactly one entry with 1, whereas in Y we can have several such entries. Furthermore, $y_{s,c} = 0$ implies $z_{s,c} = 0$. We call Z the committed categorization, and Y the uncommitted categorization. In the case of multiple mappings, we have many different Z-matrices that are compat-

ible with Y (meaning that each row in Z has sum 1, and $y_{s,c} = 0$ implies $z_{s,c} = 0$) and are unobservable. Then, the likelihood of the observation (i.e., read segments) $\mathcal{L}(\rho)$ is defined as follows:

$$\mathcal{L}(\rho) = \prod_{s} \sum_{c} y_{s,c}\rho_c.$$

However, this maximum likelihood solution for $\mathcal{L}(\rho)$ cannot be obtained in closed form. Hence, we apply the following EM algorithm to determine the maximal likelihood estimates $\hat{\rho}$.

E-Step

Let $\rho^{(t)}$ be the vector of abundance estimates $\rho_c^{(t)}$ in round t of the EM-algorithm. The E-step consist of the determination of the expected values for the hidden variables:

$$\begin{aligned} E[z_{s,c} \mid Y, \rho^{(t)}] &= Pr[z_{s,c} = 1 \mid \rho^{(t)}, Y] \\ &= \frac{\rho_c^{(t)}}{\sum_{c'} y_{s,c'} \rho_{c'}^{(t)}} \end{aligned} \quad (1)$$

Note that we are not only interested in determining the abundances of the CRLs, but also in the likelihood that a read segment s is from a CRL c, i.e., in $Pr[z_{s,c} = 1 \mid \hat{\rho}, Y]$, for which we can use the values calculated in equation (1) in the last E-Step of the EM-algorithm. From these likelihoods, we can calculate the probability $Pr[(s,s') \in c \leftrightarrow c']$ that a chimeric read $..s..s'..$ is an interaction between CRLs c and c':

$$Pr[(s,s') \in c \leftrightarrow c'] = Pr[z_{s,c} = 1 \mid \hat{\rho}, Y] Pr[z_{s',c'} = 1 \mid \hat{\rho}, Y]$$

Note that the relative abundance of the transcript does not influence this probability, as we consider only the read segment s (resp. s') and $\sum_c y_{s,c} Pr[z_{s,c} = 1 \mid \hat{\rho}, Y] = 1$ (resp. $\sum_c y_{s',c} Pr[z_{s',c} = 1 \mid \hat{\rho}, Y] = 1$.

M-Step

The M-step is simply the maximum likelihood estimates, given the hidden values z:

$$\rho_c^{(t+1)} = \frac{\sum_s z_{s,c}^{(t+1)}}{N} \quad (2)$$

We repeat the E and M steps until the sum of differences between the relative abundances of CRLs in 2 consecutive iterations is not higher than a user-defined value ϵ i.e., $\sum_{c=1}^{K} |\rho_c^{(t+1)} - \rho_c^t| \leq \epsilon$. The default value for ϵ that we use is $1e^{-5}$. The expression levels of the CRLs are reported in Transcripts Per Million (TPM). Calculation of TPM is explained in the supplementary section S3.

Extraction and hybridization of chimeric loci

In this final step, we extract the two most probable chimeric arms for each chimeric read along with their alignment and sequence information. If a GTF file is provided, we annotate the interacting regions with gene ids, symbols, biotypes, etc. For protein-coding genes, the biotypes are further categorized into 5′ UTR, coding sequence, and 3′ UTR. For hybridization of chimeric arms we use `IntaRNA` [38]. Occasionally, the real interaction is in the vicinity of the sequenced arms. For this reason, we hybridize the reference loci sequences from the output instead of the aligned read sequences. These reference loci are merged from multiple overlapping alignments and already contain some context of mapped arm locations.

Visualization framework

Motivation

`ChiRAViz` visualizer is developed in JavaScript (JS) to summarize, filter, and visualize the output of `ChiRA`. The output of `ChiRA` is a tabular file with each record containing interacting positions of a read on the reference with their annotation information (in case GTF was provided during the analysis) like gene ids, biotypes, gene symbols, alignment information, etc. Each such record contains more than 30 columns and depending on the library size and complexity of the interactome there can be millions of records in a single output file. Working with such large data is hard especially extracting elements of significant interactions from its native tabular form. Therefore, to summarize the complete data, a visualizer is needed where information can be filtered and shown in the form of various charts that are easier to understand.

Datatype

The visualizer is integrated into Galaxy as a native visualization for chira.sqlite datatype. Using a database allows SQLite queries to be formulated and executed to fetch a subset of data by applying filters on its columns.

User Interface

The user interface (UI) of the visualizer is created using JS and multiple JS related packages such as UnderscoreJS, Bootstrap, and jQuery. UnderscoreJS methods are used for better manipulation of JS arrays and dictionaries. Bootstrap is used for styling the UI and jQuery for document object model manipulation and asynchronous methods to fetch data from the database file.

Results and Discussion

Data

We applied `ChiRA` on a custom-made benchmark data to assess the performance, and on published RNA-RNA interactome and structurome datasets to validate the approach and showcase the functionality.

Benchmark data

Based on the benchmark data provided by the `CLAN` publication, we produced our benchmark data to test the performance of `ChiRA`. The reads were unchanged but we modified the reference sequences. The reads imitate CLASH experimental data. Each read is a direct fusion of (sub)sequences of human hg38 miRBase [39] mature miRNAs and a random TargetScan [40] target sequence (i.e., the target sequence is not necessarily a true target of this miRNA). The reads are in FASTA format and contain 1 million reads per sample. There are 5 different samples of simulated chimeric reads, each containing a specific chimeric arm length (10, 12, 15, 18, and 20). These datasets are called *noInsert* data. There is a second set of data with the same arm lengths but a random 5 nucleotide sequence inserted either between or at the ends of the arms of each chimeric read. This dataset is called *Insert* data. In both cases, if the reference miRNA or reference TargetScan target is shorter than the arm length, the whole reference sequence was used.

As a reference database, we used miRBase mature miRNAs together with TargetScan target sites. The reference sequences used in the `CLAN` publication were very short in length with a mean length of 21 nt for miRNAs and 14 nt for target reference sequences. Using those short TargetScan targets only as a reference is not realistic. Moreover, the TargetScan target sequences were predicted by a computational approach and generally not used as a reference database. With very short

target sequences it is fairly easy for the aligners to map the reads to exact locations uniquely. Adding some context poses an additional challenge to the aligners and results in multi or wrong alignments. Hence, to test the potential of our workflow on more complicated and near real-world reference sequences, we modified the target reference data as following. First, we sorted all the target genomic regions and then extended each region until the next target region is within a 200 nt range. In the end, we extracted the sequences of these positions. This procedure results in target sequences of various lengths. Similar to the real reference database, there is also a fair chance of having multiple target sites on a single reference sequence. In the original CLAN benchmark data, there were duplicate reference sequences. These were coming from the same duplicated targets of different miRNAs. All these duplicated reference sequences have been removed from our benchmark data.

Published data

To show the functionality of ChiRA, we applied ChiRA also on published datasets. We analyzed human miRNA interactome data from CLASH and mouse interactome data from CLEAR-CLIP protocols. For RNA-RNA interactome and structurome data, we used lymphoblastoid cells ployA, human ES polyA, and human RA polyA samples from SPLASH and mouse ES, human HEK293T samples from PARIS protocols. For CLASH and CLEAR-CLIP we built the reference databases as explained in the methods from their respective articles. For SPLASH and PARIS datasets we used the cDNA sequences of *hg38* and *mm10* genome builds from Ensembl revision 100. Summary of published data and its processing is explained in the supplementary section S2.

Performance on the benchmark data

We chose the same terminology as in the CLAN article to categorize the reads based on alignment types. An "arm" is the one of chimeric read segments, and an "agreed arm" is an arm that has an alignment with at least 80% overlap on correct reference location. The categories are defined as follows: "perfect" - has both uniquely mapped agreed arms; "partial_multi" - has a uniquely mapped agreed arm and a multi-mapped agreed arm; "both_multi" - both arms are multi-mapped agreed arms; "partial_wrong" - has one uniquely mapped agreed arm, and the other one is wrongly mapped; "both_wrong" - both arms are wrongly mapped; "partial_miss" - has one mapped and one unmapped arm; "both_miss" - both arms are unmapped. We carried out two separate runs of ChiRA using BWA-MEM and CLAN aligners. Figures 2 and 3 show their respective performances. Each bar in the plot represents the result of one of the two modes *naive* or *chira*. The *naive* mode is running the alignment tool (BWA-MEM or CLAN depending on the run) on the single reference database obtained by concatenating both mature miRNAs and TargetScan targets together, resulting in a gapped alignment. The reads are then directly categorized into one of the 7 above mentioned categories. When using BWA-MEM in *naive* mode, we considered only the longest alignment for each arm. In cases of multiple longest alignments, we considered all of them. In the *chira* mode, ChiRA workflow with the corresponding aligner was used to obtain the results. In this mode, we used a split reference, i.e., the two separate reference databases for mature miRNAs and target sequences. We also enabled the CRL creation while quantifying. The bars are then grouped horizontally based on the arm lengths and then furthermore grouped by whether the reads contain inserts or not.

The most challenging cases are with arm lengths of 10 and 12 nt. Being very short sequences, these cases tend to result in a lot more multi-mappings than the others. In *naive* mode, for an arm length of 10 nt there are a negligible number of "perfect" reads. The *chira* mode could detect some "perfect" reads but they are still less than 10% in any case. Considering the short length of the arms, it is clear that these generally map to multiple or wrong locations. For an arm length of 12 nt, there is more than 2.5-fold increment in "perfect" reads from *naive* to *chira* mode. At this arm length there is still not at an acceptable number of "perfect" reads except for CLAN aligner on *noInsert* data. The percentages of "perfect" reads are consistently around 70% for arms of lengths 15 nt and above for both the aligners in *naive* mode. This observation indicates that the sequenced RNA fragments must be at least 15 nt long to be uniquely identified at an acceptable rate. Despite being a chimeric read aligner, CLAN produced a significant amount of ambiguous "partial_multi" and "both_multi" alignments in *naive* mode (Figure 3). ChiRA sensitive mapping combined with CRL quantification is good at picking the correct alignments. For this reason, in *chira* mode there is at least 10% more "perfect" reads in all samples.

There is a decreasing trend in "perfect" reads for CLAN-based results on reads of lengths 15 to 20 nt with inserts, whereas it is more stable for BWA-MEM-based results. As this trend can also be seen in *naive* mode, it is likely more of a flaw of the aligner than ChiRA processing. For BWA-MEM-based alignments we consider an arm to be unmapped if it has no alignment on the sense strand. For this reason, there are many reads in "partial_miss" and "both_miss" categories for BWA-MEM-based results even though there might be wrong alignments on the anti-sense strand.

For reads with shorter arms, even with very sensitive alignment settings, both aligners struggled to map to correct locations. Hence, we suggest tweaking the alignment settings of the aligners to capture read segments of at least 15 nt long. Shorter alignments often tend to be from ambiguous or wrong locations and eventually lead to false-positive interactions.

Inferring common read loci significance from published data

For the analysis of all published datasets, we used BWA-MEM to map the reads to reference databases and enabled CRL creation. From the process of creating CRLs, it is noticeable that the loci of a CRL share a common reference sequence. In this section we show that CRLs are not just random groups but have high sequence identity and genes associated with the loci of a CRL implicate common annotations and functions.

CRLs and sequence identity

To determine the extent of the similarity among the CRL member loci, we computed the sequence identities. Each locus within a CRL is unique and does not contain any duplicate regions from gene isoforms. While running the workflow we used the default value of 0.7 for the option -crl_share_threshold. With this option loci having at least 70% of reads in common are grouped into a CRL. First, for each CRL we computed all pairwise global alignments among the loci using Biopython module pairwise2 [41] with default alignment parameters. With no gap or mismatch penalties in default parameters, we essentially counted the number of matching bases. We then calculated the mean of pairwise sequence identities (PSI) per CRL and a final mean per sample overall CRLs normalized by the CRL size. PSI is the ratio of the alignment score to the average sequence length of the sequences. As a baseline, for each CRL size, we randomly sampled loci and computed the PSI.

Notably, with a default value of 0.7 for CRL share, we see

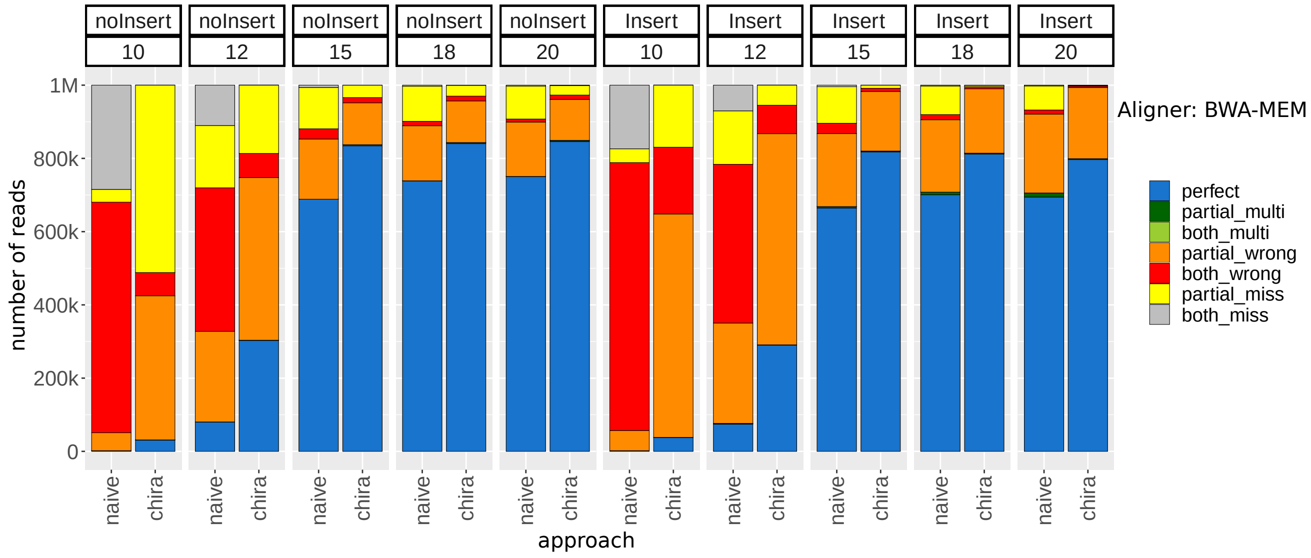

Figure 2. Performance of BWA-MEM based ChiRA compared to naive approach on benchmark data. ChiRA based results have at least 10% more perfect hits compared to *naive* mode for any arm length.

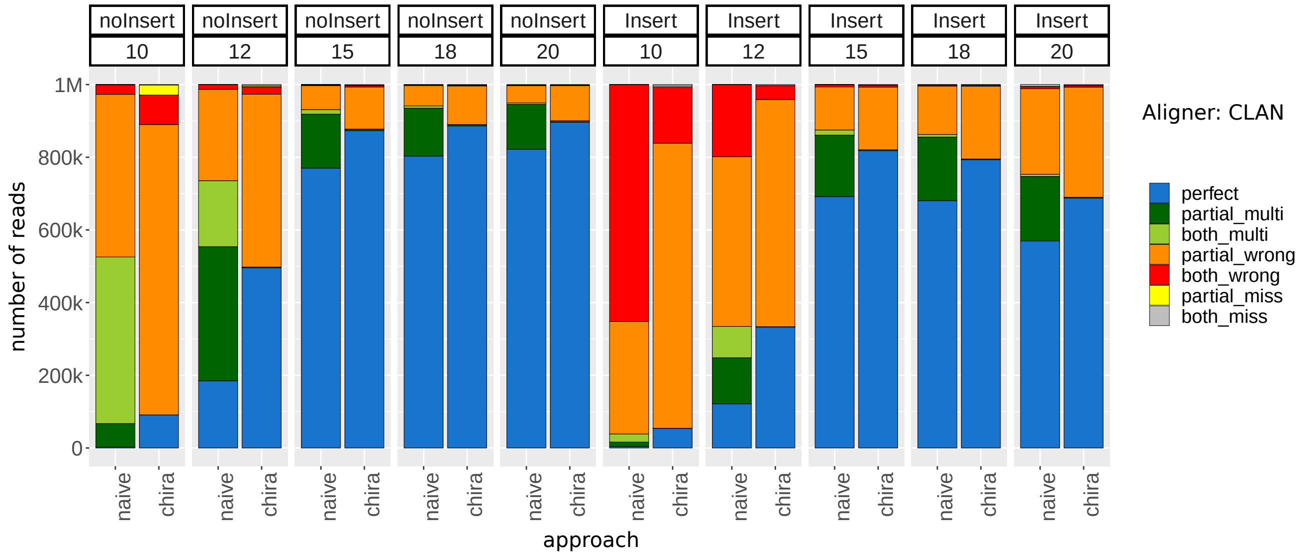

Figure 3. Performance of CLAN based ChiRA compared to naive approach on benchmark data. Being a chimeric read aligner CLAN produced less wrong hits and more multi hits that contain the true alignment. CRL-based ChiRA could pick the correct reference from the multi-mapped hits for any arm length. Note that though there are more multi-hits (green) in *naive* mode compared to *chira* mode, the origin of these reads is still uncertain.

that the PSIs among the loci within CRLs have a median of at least 90% (Figure 4) whereas the PSIs for randomly sampled loci is only around 50%. This similarity among the CRL loci is very compelling considering that the global alignment is used. It is also consistent across different sequencing protocols.

Biological relevance of CRLs

In Robert & Watson *et al.* [31], it has been shown for a hand-full of genes that the groups of genes that are consistently multi-mapped are from gene families. Similarly, here on a large scale, we analyzed if the genes that constitute the CRLs share biologically relevant information. We created an annotation database by extracting Rfam family, Ensembl protein family and KEGG pathway information from Ensembl biomart [42]. We excluded all the CRLs from the analysis that do not contain at least 2 annotated genes in the database. For each CRL, we counted the number of genes with the same protein family or the same KEGG pathway or enzyme id. We then calculated the ratio of this number to the total number of genes per CRL. In the end, we computed a weighted average over all the samples for each experimental protocol. As a control for each CRL, we randomly sampled the same number of genes out of the databases and calculated the percentage of those genes sharing a protein family or KEGG id. Figure 5 shows the boxplots for the above explained values for CRL genes and randomly sampled genes for each experimental protocol. In all cases, it is evident that for most of the CRLs gene constitution is explainable compared to random genes constitution. Although not all of the CRLs have explainable sources (for eg, CLEAR-CLIP and SPLASH), overall the genes from a CRL are more often belong to the same gene family or KEGG pathway than randomly sampled genes. Note that the CRLs are built from the short loci which are just tiny portions of the genes. But here we are evaluating them at the whole gene level to which they belong to. Though the loci are highly similar, the gene level assessment might not necessarily explain all the CRLs.

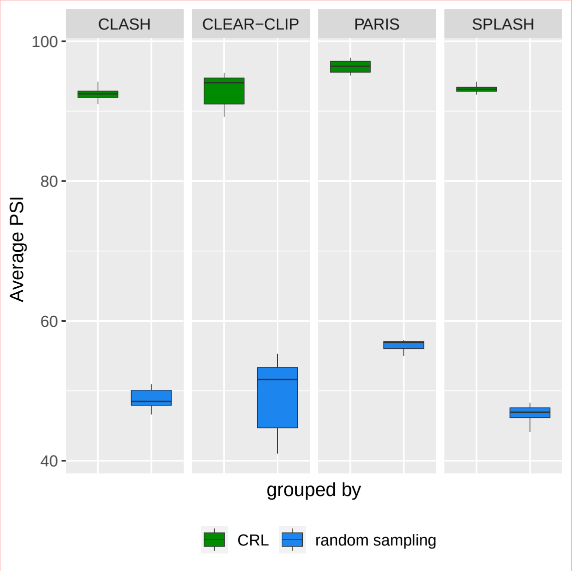

Figure 4. Boxplots showing the average pairwise sequence identities. Green boxplots represent the PSI among the member loci of the CRLs, whereas the blue boxplots show the PSIs calculated from the randomly sampled loci. The loci sequences belonging to the CRLs show higher PSI than random sampling.

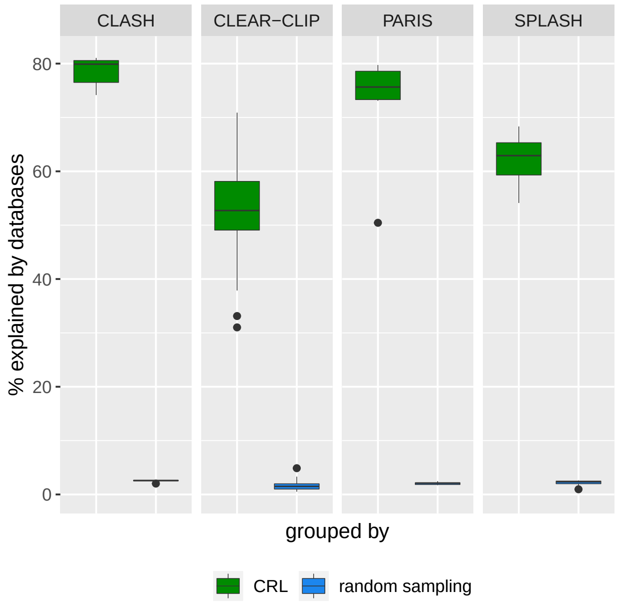

Figure 5. Validation of the CRLs from the different experimental protocols. The box plots in green show the percentage of genes belonging to the CRLs that belong to the same protein family or have a similar KEGG identifier. The blue box plots show the same information, but when the genes were randomly sampled. In all datasets, it is clear that the genes constituting CRLs are found to be related in at least one of the annotation databases.

Sensitive chimeric read detection using `ChiRA`

Finally, we tested the sensitivity of `ChiRA` in detecting interactions by analyzing all CLASH and CLEAR-CLIP mouse datasets and subsequently comparing them with the published interactions. To be consistent with the published interactions, for CLASH we considered miRNA ids with their target transcript positions and for CLEAR-CLIP miRNA ids with their target genomic positions. As we used the transcriptomic database for mapping, we ignored the intronic and intergenic target sites from CLEAR-CLIP published interactions. From `ChiRA` output, we selected chimeric reads with a final probability of at least 0.5 and the detected interacting loci that could be hybridized by `IntaRNA`. Figures 6 and 7 show Venn diagrams intersecting the published interactions and interactions predicted by `ChiRA` for CLASH and CLEAR-CLIP datasets respectively. There is a large overlap of 83% with CLASH and 73% with CLEAR-CLIP published interactions despite using different aligners. Compared to the published dataset(s), `ChiRA` on average detects three times more interactions. Given our analysis of benchmark data (Figures 2 and 3), and supported by `IntaRNA` hybridization of interacting loci, we assume that majority of these detected interactions are true positives.

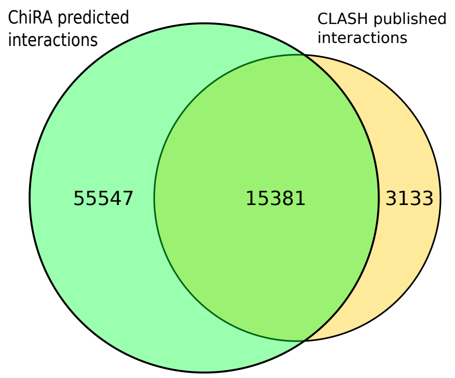

Figure 6. Number of interactions that were detected by `ChiRA` compared to published interactions in CLASH datasets

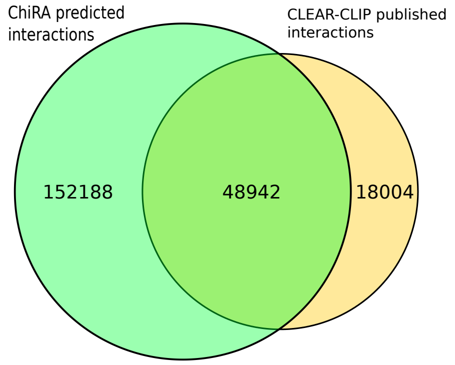

Figure 7. Number of interactions that were detected by `ChiRA` compared to published interactions in CLEAR-CLIP datasets

Visualization of chimeric reads

The visualization has three views. The first page, shown in Figure 8A displays numerous plots to summarize the complete data. Two pie charts show the RNA biotype distribution of interacting transcripts. Another pie chart shows the distribution of interactions. Moreover, there is a bar plot that lists the gene symbols of top interacting transcripts sorted in decreasing order of their respective loci expressions. At the top of the page, there are two select boxes for choosing the interacting RNA types. When an interacting pair is chosen from these select boxes, it redirects to the second page (Figure 8B) that shows all the interactions that involve these selected RNA biotypes.

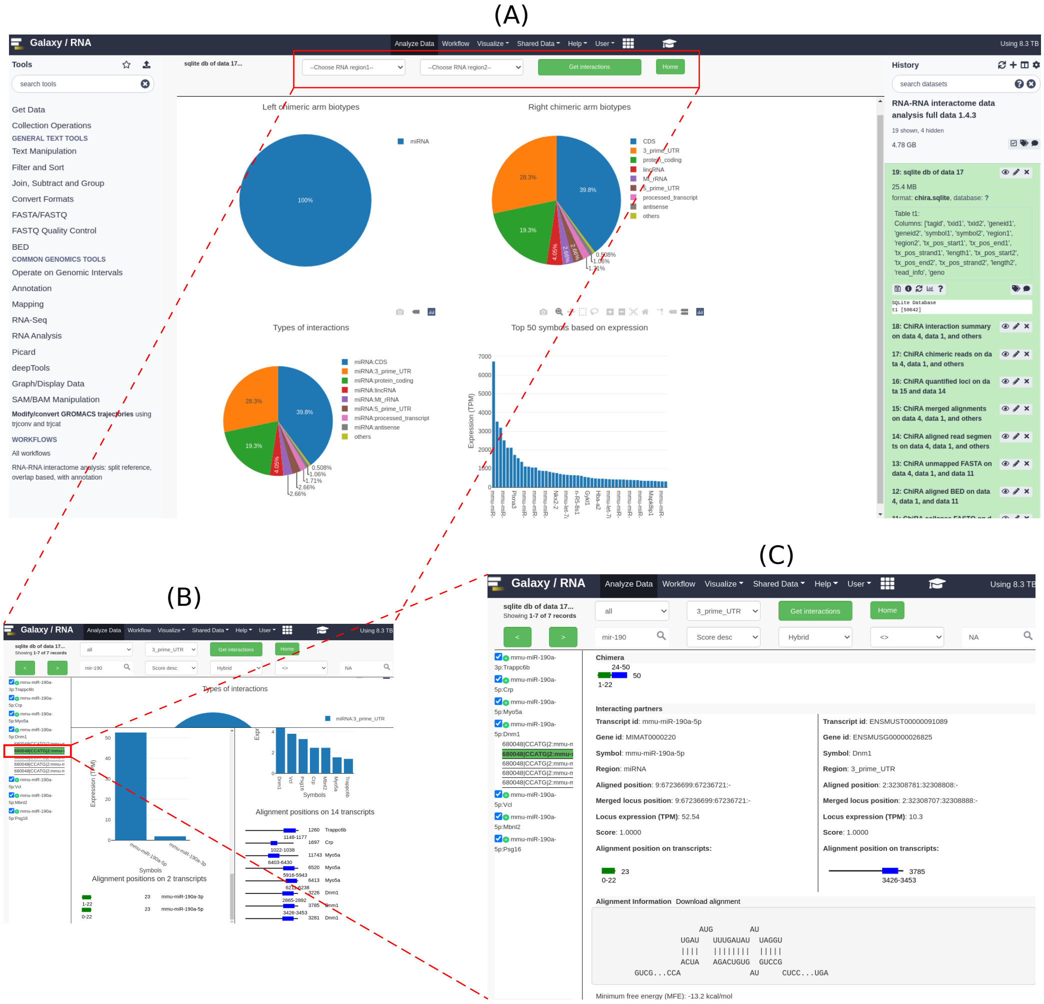

Figure 8. ChiRAViz Galaxy visualization. (A) The home page of the visualization. The plots in this page summarize the RNA biotypes of left and right chimeric arms, types of interactions, and highly abundant genes within the sample. (B) The Second page shows the interactions of selected biotypes. On this page, users can further search, sort, and filter the interactions and obtain a deep summary of filtered interactions. (C) Interaction information page that shows all the useful information like gene symbol, transcript ids, gene ids, expression level, biotypes, a depiction of interaction reference regions at transcript level, an illustration of the aligned read positions, and IntaRNA predicted hybrid

On the left, there is a list of unique combinations of gene symbols that represent unique RNA-RNA interactions. At the top of this page, there are several filters such as search and sort which facilitate data to be fetched in the desired way. All or some of these entries can be selected together and a summary can be seen in the form of pie charts, histograms, and transcript level alignment positions. The pie charts show distributions of the gene symbols and biotypes and the histograms show the distributions of alignment scores and their loci expressions. The alignment regions on each interacting transcript are also depicted with the start, end, and length of the alignment. All the selected interactions can be exported as a tab-separated file to the local computer. The pagination shown at the top left corner helps to navigate through all the interactions and displays a small number of interactions (50) at a time, which simplifies the UI. All the unique reads associated with each interaction can be seen by clicking on the "+" icon adjacent to the interactions themselves. Clicking on any of these single records displays the interaction summary page, as shown in Figure 8C. This page shows all the information related to interacting partners such as gene id, gene symbol, biotype, alignment start and end positions, transcript length, CIGAR string of the read alignment, and the expression of its corresponding locus in TPM. If there is an IntaRNA predicted hybrid, it is shown at the bottom of this page.

Integration into Galaxy framework and tutorial

Galaxy [25] has been one of the most popular resources for reproducible research. It also makes the tool usage easier by cutting off the tool dependency installation and by offering a single click tool or workflow execution. With public Galaxy servers, users also get access to huge computing resources. We integrated all of our tools into Galaxy. The whole Python suite is available through Bioconda [43] and BioContainers [44] for easy installation. Galaxy Training Network (GTN) is a Galaxy community aimed at developing the analysis-specific training material [45]. We developed training material for RNA-RNA interactome data analysis that includes a step-by-step guide of hands-on Galaxy analysis workflows with example datasets, ready-to-use Galaxy workflows, and an example Galaxy history. The training material also deals with the visualization framework. Being nicely coupled into Galaxy ecosystem ChiRA is now part of RNA workbench [46], a large comprehensive Galaxy-based web server for RNA-based research. All the data and ChiRA analysis discussed in this paper is available through RNA workbench. Figure 9 shows the ChiRA Galaxy workflow that uses split reference.

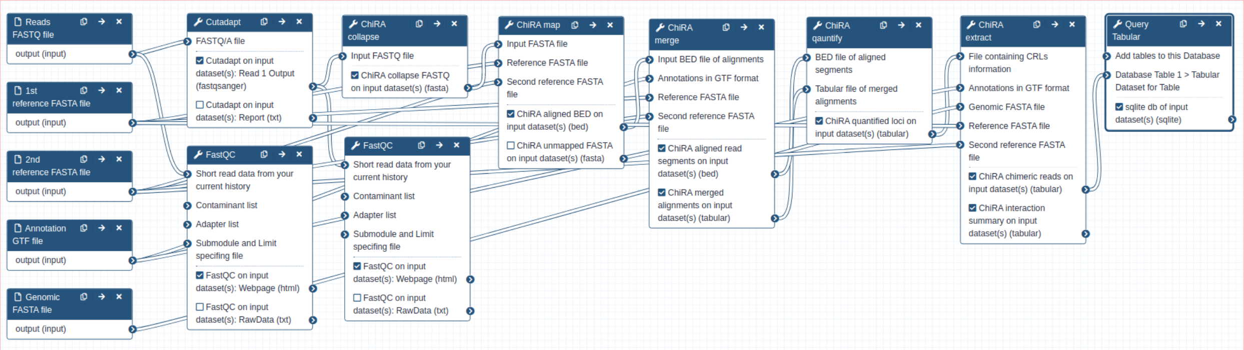

Figure 9. ChiRA Galaxy workflow. The workflow takes the FASTQ files that contain raw sequencing reads, process them, and produces a tabular and an SQLite database of interactions that are ready to be visualized by ChiRAViz.

Conclusion

In this article, we presented a comprehensive solution for RNA-RNA interactome and RNA structurome data analysis. Our method of creating CRLs from loci with consistent multi-mapped reads and quantification proved to rescue more reads from benchmark data. We also showed that the loci within a CRL have high sequence identities and the genes that constitute the CRLs originate from the same protein families or share common functional pathways revealing that it is sensible to group consistently multi-mapped loci into CRLs. To the best of our knowledge, ChiRA along with ChiRAViz is the only tool suite that makes analysis of RNA-interactome and structurome datasets easily accessible to the users through Bioconda and Galaxy.

Availability of Source Code and Requirements

- Project name: ChiRA
- Project home page: https://github.com/pavanvidem/chira
- Visualization: https://github.com/galaxyproject/galaxy/tree/dev/config/plugins/visualizations/chiraviz
- Operating system(s): Platform independent
- Programming language: Python
- Other requirements: Anaconda
- Installation: `conda install -c conda-forge -c bioconda chira`
- License: GNU GENERAL PUBLIC LICENSE Version 3
- Galaxy tool suite: https://github.com/galaxyproject/tools-iuc/tree/master/tools/chira
- Galaxy training tutorial: https://galaxyproject.github.io/training-material/topics/transcriptomics/tutorials/rna-interactome/tutorial.html
- Galaxy workflows:

  https://rna.usegalaxy.eu/u/videmp/w/rna-rna-interactome-analysis (using BWA-MEM), https://rna.usegalaxy.eu/u/videmp/w/rna-rna-interactome-analysis-using-clan (using CLAN)

- BiotoolsID: chira
- RRID: SCR_019219

Availability of supporting data and materials

The benchmark data that was used to evaluate the performance of ChiRA can be obtained from Zenodo [47].

List of abbreviations

BAM: Binary sequence Alignment/Map; BED: Browser Extensible Data; CRL: Common Read Loci; CLASH: Cross-linking Ligation And Sequencing of Hybrids; GTF: Gene Transfer Format; JS: JavaScript; LIGR-Seq: LIGation of interacting RNA followed by high-throughput Sequencing; PARIS: Psoralen Analysis of RNA Interactions and Structures; PSI: Pairwise Sequence Identities; SAM: Sequence Alignment/Map; SPLASH: Sequencing of Psoralen crosslinked, Ligated, And Selected Hybrids; UI: User Interface; UTR: Untranslated Region.

Ethical Approval

Not applicable

Consent for publication

Not applicable

Competing Interests

The authors declare that they have no competing interests.

Funding

This work was supported by the German Research Foundation (DFG) grant eCLASH: Definition des Interactomes kleiner RNA [2168/14-1 awarded to R.B.] and the DFG-funded Collaborative Research Centre 992 Medical Epigenetics [SFB 992/1 2012 awarded to R.B.]. The article processing charge was funded by the Baden-Württemberg Ministry of Science, Research and Art and the University of Freiburg in the funding programme Open Access Publishing.

Author's Contributions

Pavankumar Videm implemented the ChiRA tool suite, integrated into Galaxy, created training material, analyzed the data and wrote the major portion of the manuscript. Anup Kumar developed the ChiRAViz Galaxy visualization and was involved

in writing corresponding sections of the manuscript. Björn Andreas Grüning and Oleg Zharkov supported in galaxy integration and deployment. All the authors were involved in reviewing the manuscript.

Acknowledgements

We thank Sita J. Saunders for fruitful discussions and support in writing biological introduction. We are grateful to Michael Uhl for thorough revision of the manuscript and his constructive comments. The authors also acknowledge the support of the Freiburg Galaxy Team: Prof. Rolf Backofen, Bioinformatics, University of Freiburg, Germany funded by Collaborative Research Centre 992 Medical Epigenetics (DFG grant SFB 992/1 2012) and German Federal Ministry of Education and Research (BMBF grant 031 A538A de.NBI-RBC).

References

1. Ambros V. The functions of animal microRNAs. Nature 2004;431(7006):350–355.
2. Henras AK, Dez C, Henry Y. RNA structure and function in C/D and H/ACA s (no) RNPs. Current opinion in structural biology 2004;14(3):335–343.
3. Bartel DP. MicroRNAs: genomics, biogenesis, mechanism, and function. cell 2004;116(2):281–297.
4. Mattick JS, Makunin IV. Non-coding RNA. Human molecular genetics 2006;15(suppl_1):R17–R29.
5. Plotnikova O, Skoblov M. Efficiency of the miRNA-mRNA interaction prediction programs. Molecular Biology 2018;52(3):467–477.
6. Helwak A, Kudla G, Dudnakova T, Tollervey D. Mapping the human miRNA interactome by CLASH reveals frequent noncanonical binding. Cell 2013;153(3):654–665.
7. Moore MJ, Scheel TKH, Luna JM, Park CY, Fak JJ, Nishiuchi E, et al. miRNA–target chimeras reveal miRNA 3'-end pairing as a major determinant of Argonaute target specificity. Nat Commun 2015 nov;6:8864. http://www.nature.com/doifinder/10.1038/ncomms9864.
8. Lu Z, Zhang QC, Lee B, Flynn RA, Smith MA, Robinson JT, et al. RNA duplex map in living cells reveals higher-order transcriptome structure. Cell 2016;165(5):1267–1279.
9. Aw JGA, Shen Y, Wilm A, Sun M, Lim XN, Boon KL, et al. In vivo mapping of eukaryotic RNA interactomes reveals principles of higher-order organization and regulation. Molecular cell 2016;62(4):603–617.
10. Sharma E, Sterne-Weiler T, O'Hanlon D, Blencowe BJ. Global mapping of human RNA-RNA interactions. Molecular cell 2016;62(4):618–626.
11. Maher CA, Kumar-Sinha C, Cao X, Kalyana-Sundaram S, Han B, Jing X, et al. Transcriptome sequencing to detect gene fusions in cancer. Nature 2009;458(7234):97–101.
12. Kannan K, Wang L, Wang J, Ittmann MM, Li W, Yen L. Recurrent chimeric RNAs enriched in human prostate cancer identified by deep sequencing. Proceedings of the National Academy of Sciences 2011;108(22):9172–9177. https://www.pnas.org/content/108/22/9172.
13. Asmann YW, Necela BM, Kalari KR, Hossain A, Baker TR, Carr JM, et al. Detection of redundant fusion transcripts as biomarkers or disease-specific therapeutic targets in breast cancer. Cancer research 2012;72(8):1921–1928.
14. Tandefelt DG, Boormans J, Hermans K, Trapman J. ETS fusion genes in prostate cancer. Endocrine-related cancer 2014;21(3):R143–R152.
15. Lewis BP, Burge CB, Bartel DP. Conserved seed pairing, often flanked by adenosines, indicates that thousands of human genes are microRNA targets. cell 2005;120(1):15–20.
16. Esteller M. Non-coding RNAs in human disease. Nature reviews genetics 2011;12(12):861–874.
17. Mendell JT, Olson EN. MicroRNAs in stress signaling and human disease. Cell 2012;148(6):1172–1187.
18. Coolen M, Bally-Cuif L. MicroRNAs in brain development and physiology. Current opinion in neurobiology 2009;19(5):461–470.
19. Pinzón N, Li B, Martinez L, Sergeeva A, Presumey J, Apparailly F, et al. microRNA target prediction programs predict many false positives. Genome research 2017;27(2):234–245.
20. Broughton JP, Pasquinelli AE. A tale of two sequences: microRNA-target chimeric reads. Genet Sel Evol 2016 dec;48(1):31. http://gsejournal.biomedcentral.com/articles/10.1186/s12711-016-0209-x.
21. Langmead B, Salzberg SL. Fast gapped-read alignment with Bowtie 2. Nature methods 2012;9(4):357.
22. Li H. Aligning sequence reads, clone sequences and assembly contigs with BWA-MEM. arXiv preprint arXiv:13033997 2013;.
23. Dobin A, Davis CA, Schlesinger F, Drenkow J, Zaleski C, Jha S, et al. STAR: ultrafast universal RNA-seq aligner. Bioinformatics 2013;29(1):15–21.
24. Travis AJ, Moody J, Helwak A, Tollervey D, Kudla G. Hyb: a bioinformatics pipeline for the analysis of CLASH (crosslinking, ligation and sequencing of hybrids) data. Methods 2014;65(3):263–273.
25. Afgan E, Baker D, Batut B, Van Den Beek M, Bouvier D, Čech M, et al. The Galaxy platform for accessible, reproducible and collaborative biomedical analyses: 2018 update. Nucleic acids research 2018;46(W1):W537–W544.
26. Martin M. Cutadapt removes adapter sequences from high-throughput sequencing reads. EMBnet journal 2011;17(1):10–12.
27. Zhong C, Zhang S. Accurate and Efficient Mapping of the Cross-Linked microRNA-mRNA Duplex Reads. iScience 2019;18:11–19.
28. Li H, Handsaker B, Wysoker A, Fennell T, Ruan J, Homer N, et al. The Sequence Alignment/Map format and SAMtools. Bioinformatics 2009 06;25(16):2078–2079. https://doi.org/10.1093/bioinformatics/btp352.
29. Langenberger D, Bermudez-Santana C, Hertel J, Hoffmann S, Khaitovich P, Stadler PF. Evidence for human microRNA-offset RNAs in small RNA sequencing data. Bioinformatics 2009;25(18):2298–2301.
30. Holmqvist E, Wright PR, Li L, Bischler T, Barquist L, Reinhardt R, et al. Global RNA recognition patterns of post-transcriptional regulators Hfq and CsrA revealed by UV crosslinking in vivo. The EMBO journal 2016;35(9):991–1011.
31. Robert C, Watson M. Errors in RNA-Seq quantification affect genes of relevance to human disease. Genome biology 2015;16(1):177.
32. Zhang Z, Xing Y. CLIP-seq analysis of multi-mapped reads discovers novel functional RNA regulatory sites in the human transcriptome. Nucleic acids research 2017;45(16):9260–9271.
33. Van Nostrand EL, Pratt GA, Yee BA, Wheeler EC, Blue SM, Mueller J, et al. Principles of RNA processing from analysis of enhanced CLIP maps for 150 RNA binding proteins. Genome biology 2020;21:1–26.
34. Teng M, Love MI, Davis CA, Djebali S, Dobin A, Graveley BR, et al. A benchmark for RNA-seq quantification pipelines. Genome biology 2016;17(1):74.
35. Pachter L. Models for transcript quantification from RNA-Seq. arXiv preprint arXiv:11043889 2011;.

36. Jin H, Wan YW, Liu Z. Comprehensive evaluation of RNA-seq quantification methods for linearity. BMC bioinformatics 2017;18(4):117.
37. Xing Y, Yu T, Wu YN, Roy M, Kim J, Lee C. An expectation-maximization algorithm for probabilistic reconstructions of full-length isoforms from splice graphs. Nucleic acids research 2006;34(10):3150–3160.
38. Mann M, Wright PR, Backofen R. IntaRNA 2.0: enhanced and customizable prediction of RNA–RNA interactions. Nucleic acids research 2017;45(W1):W435–W439.
39. Griffiths-Jones S. miRBase: the microRNA sequence database. In: MicroRNA Protocols Springer; 2006.p. 129–138.
40. Agarwal V, Bell GW, Nam JW, Bartel DP. Predicting effective microRNA target sites in mammalian mRNAs. elife 2015;4:e05005.
41. Cock PJ, Antao T, Chang JT, Chapman BA, Cox CJ, Dalke A, et al. Biopython: freely available Python tools for computational molecular biology and bioinformatics. Bioinformatics 2009;25(11):1422–1423.
42. Kinsella RJ, Kähäri A, Haider S, Zamora J, Proctor G, Spudich G, et al. Ensembl BioMarts: a hub for data retrieval across taxonomic space. Database 2011;2011.
43. Grüning B, Dale R, Sjödin A, Chapman BA, Rowe J, Tomkins-Tinch CH, et al. Bioconda: sustainable and comprehensive software distribution for the life sciences. Nature methods 2018;15(7):475–476.
44. da Veiga Leprevost F, Grüning BA, Alves Aflitos S, Röst HL, Uszkoreit J, Barsnes H, et al. BioContainers: an open-source and community-driven framework for software standardization. Bioinformatics 2017;33(16):2580–2582.
45. Batut B, Hiltemann S, Bagnacani A, Baker D, Bhardwaj V, Blank C, et al. Community-driven data analysis training for biology. Cell systems 2018;6(6):752–758.
46. Fallmann J, Videm P, Bagnacani A, Batut B, Doyle MA, Klingstrom T, et al. The RNA workbench 2.0: next generation RNA data analysis. Nucleic acids research 2019;47(W1):W511–W515.
47. Videm P, Benchmark data used in the evaluation of ChiRA tool-suite. Zenodo; 2020. https://doi.org/10.5281/zenodo.4289365.

Click here to access/download
Supplementary Material
GIGA-D-20-00250_revised_supplement.pdf
