# Supplementary material for: ChiRA: an integrated framework for chimeric read analysis from RNA-RNA interactome and RNA structurome data
Source: Gigascience. 2021 Jan 29;10(2):giaa158. doi: 10.1093/gigascience/giaa158 (PMC7844879; doi:10.1093/gigascience/giaa158)
Supplement: giaa158_Supplemental_File [file giaa158_supplemental_file.pdf]

ChiRA: an integrated framework for Chimeric Read Analysis from RNA-RNA interactome and RNA structurome data

Pavankumar Videm[1], Anup Kumar[1], Oleg Zharkov[1], Björn Andreas Grüning[1], and Rolf Backofen[1,2]

[1]Bioinformatics Group, Department of Computer Science, University of Freiburg, Georges-Koehler-Allee 106, 79110 Freiburg, Germany
[2]Signalling Research Centres BIOSS and CIBSS, University of Freiburg, Schaenzlestr. 18, 79104 Freiburg, Germany

S1 Glossary of terms

This section gives a brief into the terminology used in the manuscript.

- *Read arm*: An aligned portion of the read.
- *Singleton read*: A read with only one arm. A single arm may multi-map to different positions on the reference.
- *Chimeric read*: A read with at least two non-overlapping arms. Each of these arms called as a *chimeric arm*.
- *Read segment*: A region on the read assembled by merging highly overlapping arms.
- *Expressed locus*: A region on the reference built from overlapping alignments. This can be achieved by a simple overlapping method or `blockbuster`'s Gaussian approximation of read coverage.
- *Common Read Loci*: A set of expressed loci that share most of their multi-mapped reads.

S2 Data and pre-processing

We applied `ChiRA` on CLASH, CLEAR-CLIP, PARIS and SPLASH datasets. Altogether 46 samples were analyzed using `ChiRA`. The details of the samples are in Table S1.

Protocol	Samples (GEO or SRA ids)	#	Organism	Characteristics
CLASH	GSM1219487, GSM1219488, GSM1219489, GSM1219490, GSM1219491, GSM1219492	6	human	Flp-In T-REx 293
CLEAR-CLIP	GSM1881516, GSM1881517, ..., GSM1881541	26	mouse	cortex CLEAR-CLIP
PARIS	GSM1917755, GSM1917756, GSM1917757, GSM1917758, GSM1917759, GSM1917760	6	human & mouse	HEK293T (3), mouse ES(3)
SPLASH	SRR3404939, SRR3404940, SRR3404941, SRR3404942, SRR3404943, SRR3404926, SRR3404927, SRR3404928	8	human	Lymphoblastoid Cells PolyA (4), H1 hES PolyA (2), H1 hES treated with retinoic acid Poly A (2)

Table S1: Summary of the samples analyzed using `ChiRA`

The whole analysis of the above mentioned samples was carried out on RNA workbench [1]. We used `fastq-dump` Galaxy tool [2] to fetch the fastq files from the sequence read archive (SRA) database. Then we removed adapters and low quality ends using `cutadapt` [3]. We followed the instructions from the papers and gene expression omnibus sample pages to reproduce the pre-processing step. For CLASH, we used 5′ adapter - ACACGACGCTCTTCCGATCT and 3′ adapter - TGGAATTCTCGGGTGCCAAGG. For the preprocessing of the CLEAR-CLIP data, we used 5′ adapter - NNNNAGGGAGGACGATGCGG and 3′ adapter - GTGTCAGTCACTTCCAGCGG. For PARIS datasets, 3′ adapter - AGATCGGAAGAGCGGTTCAG was used. The SPLASH samples in the SRA were already processed, hence we used them as they are. We filtered out the reads shorter than 16 nucleotides (*–minimum-length 16*) and trimmed bases from read ends with a Phred quality score less than 20 (*–quality-cutoff 20*). For all CLEAR-CLIP samples a 5 bases long 5′ degenerate linker, for CLASH GSM1219491 sample a 8nt long barcode, and for GSM1219492 sample a 9nt long barcode were stripped while deduplicating using `ChiRA-collapse` tool (*–umi_len N*).

S3 Calculation of Transcripts per Million

A the end of the EM algorithm, for each CRL c, we re-calculate the absolute abundances of as $\rho'_c = \sum_s Pr[z_{s,c} = 1|\hat{\rho}, Y]$. of abundance of c to the length of c in kilobases. This measure is generally known as reads per kilobase (RPK). As each CRL is a set of expressed loci, a CRL cannot have a single length. Hence we take the median of the lengths of loci l belong to the CRL c as the length of the CRL.

$$RPK_c = 10^3 \frac{\rho'_c}{\text{median}_{l \in c}\{length(l)\}}$$

Then transcripts per million calculated as relative RPK of a CRL compared to the total RPK of the sample in millions.

$$TPM_c = 10^6 \frac{RPK_c}{\sum_{c'} RPK_{c'}}$$

S4 Data availability

We carried the whole `ChiRA` based analysis on the RNA workbench. Hence all the data can easily be accessible and each parameter of all the tool runs can be traced back. Table S2 contains the links to Galaxy histories and their corresponding workflows used. The description of the parameters that were changed from the defaults are described in Section S2. All the workflows and histories can be imported and adapted.

**Data**	**History**	**Workflow**
CLASH	https://rna.usegalaxy.eu/u/videmp/h/clash-analysis	https://rna.usegalaxy.eu/u/videmp/w/chira-clash-clearclip
CLEAR-CLIP	https://rna.usegalaxy.eu/u/videmp/h/clear-clip-analysis	https://rna.usegalaxy.eu/u/videmp/w/chira-clash-clearclip
PARIS	https://rna.usegalaxy.eu/u/videmp/h/paris-analysis	https://rna.usegalaxy.eu/u/videmp/w/paris-analysis
SPLASH	https://rna.usegalaxy.eu/u/videmp/h/splash-analysis	https://rna.usegalaxy.eu/u/videmp/w/splash-analysis
Benchmark using `BWA-MEM`	https://rna.usegalaxy.eu/u/videmp/h/benchmark-chira-bwa	https://rna.usegalaxy.eu/u/videmp/w/benchmark-chira-bwa
Benchmark using `CLAN`	https://rna.usegalaxy.eu/u/videmp/h/benchmark-chira-clan	https://rna.usegalaxy.eu/u/videmp/w/benchmark-chira-clan

Table S2: Links to the analysis histories and the wokflows

S5 CRL validation

The complete sample-wise CRLs information is given in Table S3. The column description is as follows. PSI: column shoes the average percentage sequence identity among the loci within the CRLs of each sample. #qualified CRLs: Number of CRLs per sample used in validating CRLs. A qualified CRL must contain at least 2 distinct genomic loci that associate with different genes. The genes that are associated with the loci within a the associated genes must exist in the annotation. %explained by databases: Average percentage of genes per CRL that are in agreement with the Ensembl protein family or kegg pathways information. #reads involved: Number of unique reads that are involved in CRLs. This is the number of reads that are possibly rescued by creating CRLs. Note that these are the deduplicated reads. Compared to these deduplicated reads, the number of initial sequenced reads that resulted in these can sometimes be in orders of magnitude.

Protocol	Sample	APSI	#qualified CRLs	%explained by databases	#reads involved
CLASH	GSM1219487	94.29	894	74.15	68724
	GSM1219488	93.20	599	79.44	53078
	GSM1219489	92.62	675	75.53	81176
	GSM1219490	92.33	1763	80.36	227305
	GSM1219491	90.82	1075	81.06	295311
	GSM1219492	90.75	979	80.06	272882
CLEAR-CLIP	GSM1881516	92.43	269	62.61	23813
	GSM1881517	88.99	1026	33.13	42469
	GSM1881518	91.77	330	55.94	26294
	GSM1881519	91.07	318	51.32	24299
	GSM1881520	91.24	211	58.48	19220
	GSM1881521	94.35	255	70.90	17943
	GSM1881522	91.07	318	51.32	24299
	GSM1881523	95.32	262	55.25	15475
	GSM1881524	94.83	113	57.10	5546
	GSM1881525	90.72	838	31.02	34595
	GSM1881526	90.39	289	43.11	18574
	GSM1881527	89.50	668	37.87	28075
	GSM1881528	90.90	316	51.49	23608
	GSM1881529	90.08	479	51.47	40234
	GSM1881530	88.67	847	39.70	28228
	GSM1881531	95.60	113	58.81	3366
	GSM1881532	94.88	216	49.24	5280
	GSM1881533	95.15	149	59.31	5972
	GSM1881534	94.45	142	51.52	7072
	GSM1881535	93.98	135	53.92	3827
	GSM1881536	95.58	186	49.02	5130
	GSM1881537	94.75	165	45.85	6789
	GSM1881538	94.72	156	56.31	6449
	GSM1881539	94.92	90	59.93	7252
	GSM1881540	94.44	112	64.49	6778
	GSM1881541	95.16	111	54.77	7955
PARIS	GSM1917755	95.27	523	77.33	194356
	GSM1917756	95.66	584	73.98	177179
	GSM1917757	96.14	1505	50.44	394708
	GSM1917758	97.64	650	79.75	89945
	GSM1917759	96.94	709	73.09	141218
	GSM1917760	97.23	632	79.01	129261
SPLASH	SRR3404943	92.65	866	65.63	129261
	SRR3404926	93.32	324	63.48	108724
	SRR3404927	93.14	792	65.21	182786
	SRR3404928	93.71	343	60.00	83129
	SRR3404939	92.37	618	54.12	211750
	SRR3404940	92.89	592	68.33	176528
	SRR3404941	94.20	50	62.35	11714
	SRR3404942	93.12	201	57.25	125647

Table S3: Sample-wise summary of the numbers related to Figures 4 and 5

References

[1] J. Fallmann, P. Videm, A. Bagnacani, B. Batut, M. A. Doyle, T. Klingstrom, F. Eggenhofer, P. F. Stadler, R. Backofen, and B. Grüning, "The rna workbench 2.0: next generation rna data analysis," *Nucleic acids research*, vol. 47, no. W1, pp. W511–W515, 2019.

[2] R. Leinonen, H. Sugawara, and M. S. and, "The sequence read archive," *Nucleic Acids Research*, vol. 39, pp. D19–D21, nov 2010.

[3] M. Martin, "Cutadapt removes adapter sequences from high-throughput sequencing reads," *EMBnet. journal*, vol. 17, no. 1, pp. 10–12, 2011.
