# Supplementary material for: ChiRA: an integrated framework for chimeric read analysis from RNA-RNA interactome and RNA structurome data
Source: Gigascience. 2021 Jan 29;10(2):giaa158. doi: 10.1093/gigascience/giaa158 (PMC7844879; doi:10.1093/gigascience/giaa158)
Supplement: giaa158_Response_to_Reviewer_Comments_Original_Submission [file giaa158_response_to_reviewer_comments_original_submission.pdf]

Author's Response To Reviewer Comments

Close

Dear Nicole Nogoy,

First of all, we would like to thank you for considering our manuscript for the GigaScience journal. We registered our tool-suite at bio.tools and scicrunch.org. Their corresponding IDs can now be found under the “Availability of Source Code and Requirements” section. We appreciate the reviewers for their valuable suggestions and constructive comments. With careful consideration to each of the reviewers’ comments, we updated the manuscript when needed. The changes in the manuscript are highlighted in red color. Please find a point-by-point response to the reviewers’ comments below.
I am looking forward to hearing from you.
Sincerely yours,
Pavankumar Videm

Point-by-point response to the reviewer comments

REVIEWER 1:
Authors of the paper have put together an excellent workflow for RNA-interactome data analysis. Making the workflow available in Galaxy shows a great effort by authors to make it available for a wider audience. ChiRAViz is also a great addition to the work to visualise the results within Galaxy.
I am not an expert to judge the biological aspects of work, however, authors have tried their best to explain every aspect of the workflow in detail. I am reviewing the tool/software and visualisation aspects of the tool and manuscript.
Here, authors have put in a lot of effort to make their work available to a wider audience by making source code available from GitHub and installation from Conda package management system as well the whole workflow and intermediary tools as available in Galaxy public instance.
Authors also prepared Galaxy training material with test data and step by step guide to execute the workflow.
Following minor changes to manuscript and workflow will be good:

1. Authors have not provided a direct link to the workflow configuration file (.ga) for users to download and execute in local Galaxy, which will also allow tailoring workflow by swapping tools if needed. Though I managed to find workflow source code from Galaxy training material.

Authors’ response: We updated the links to the workflows in the section “Availability of source code, workflow, and training material”. Now they can be saved onto a local computer or imported into other Galaxy user accounts.

2. When running ChiRA, as a Galaxy workflow aligner option in ChiRAmap can only be changed in workflow editor view, it may be worthwhile to look into an option which allows changing aligner in workflow run view.

Authors’ response: The aligner is implemented as a conditional parameter in Galaxy. This type of parameter can not be changed during runtime as it could change inputs as well. This is the current limitation of the Framework and discussed in this issue: https://github.com/galaxyproject/galaxy/issues/4528 To make it more convenient for the users, we created 2 separate workflows using BWA-MEM and CLAN.

3. I have run the workflow a couple of times and the output format for SQLite database is set to .sqlite, it needs to be converted into chira.sqlite to enable visualisation plugin. The format should have been set to chira.sqlite.

Authors’ response: It is definitely a good idea to produce chira.sqlite so that it is readily usable by the visualization. The workflow now produces chira.sqlite by default.

4. Also, a Galaxy workflow overview image in the manuscript would be a good addition.

Authors’ response: Thank you for the suggestion. We added a figure (Figure 9) of the Galaxy workflow and cited it in the section “Integration into Galaxy framework and tutorial”.

5. In manuscript, section 'Visualization of chimeric reads' explains the visualisation well, but intext reference to figure and legend is missing which will help the reader to navigate while reading the text. The resulting SQLite database has one large table, I would suggest splitting the database into separate tables to make it more scalable and also potentially reusable by other tools.

Authors’ response: Thank you for your suggestion. We added references to each sub-figure based on the visualization page that is being explained. We also adapted the text to follow the steps in Figure 8. It is not necessary to split the table (horizontally) into multiple smaller tables as we provide table filtering and export features in the visualization (ChiraViz), which enables researchers and users to filter out smaller tables based on multiple parameters such as RNA biotypes, score, free energy, etc. These smaller tables can be exported/downloaded in a local machine and can be used as input files for different tools. Splitting the table vertically, which is storing related columns in different tables, would require joining all these tables (using join statements) during query time and also storing few identifier columns as foreign keys. This approach will significantly increase the query time. Therefore, we do not split the table into multiple tables.

REVIEWER 2:
The manuscript entitled ChiRA: an integrated framework for Chimeric Read Analysis from RNA-RNA interactome and RNA structurome data describes an approach for identifying RNA-RNA interactions from sequencing experiments that produce chimeric reads. The authors describe an alignment and quantification approach, including a new method to group reference loci to improve performance. The authors evaluate their approach using real published datasets and benchmark datasets. A notable strength of the manuscript is the availability of the ChiRA tools, a tutorial, and a workflow as part of Galaxy. These resources allow someone to easily reproduce the analyses presented in the manuscript or implement the ChiRA method for their own data. Overall the manuscript addresses a unique challenge, and the resources presented would be useful for anyone who generates or analyzes chimeric sequencing datasets.
I recommend this manuscript for publication after minor revisions, which are described below.

Introduction

1. The authors state that “ To support computational methods, several transcriptome-wide experimental protocols have been developed recently to detect both inter- and intra-molecular RNA structure [6, 7, 8, 9, 10]. Although, these protocols vary in their application-specific details, they currently all involve ligating the two RNA interaction partners together and subsequently sequencing the resulting chimeric RNA molecules using high-throughput-sequencing technology. ” (page 1). To be more comprehensive about approaches for detecting RNA structure, I encourage the authors to reference other types of protocols, e.g. approaches that use structure-specific enzymes to mark single- and double-stranded RNAs (e.g. PARS, DOI: 10.1038/nature09322).

Authors’ response: Although the reviewers' suggestion to include PARS like protocols make it comprehensive, we did not add them for the following reason. PARS data could only be integrated to improve the accuracy of the structure prediction as it has no information about base-pair complementarity. However, it is rarely the case to have PARS and structurome data (like SPLASH or PARIS) from the same experiment. Thus, we believe that it is at best a minor improvement in our setting but may also introduce noise. Therefore, we assume that referring these methods may bring more confusion to the readers.

2. It would strengthen the motivation of the work and provide broader interest if the authors mentioned

how their approach could be applied in contexts other than identifying miRNA:mRNA interactions, e.g. to identify chimeric RNAs in cancer or structural genome rearrangements, to name a few examples.

Authors' response: We included a short text regarding this at the end of the first paragraph of the introduction.

3. The Introduction ends a bit abruptly (page 2). I suggest adding a few sentences at the end that summarise how this work addresses the challenges described at the end of the Introduction, and highlight how the work can contribute broadly to scientific research.

Authors' response: We added a few sentences at the end of the introduction to summarize the contribution of our method. We also moved some text from the second paragraph ("Existing software solutions ... identified RNA-RNA interactions) to the end of the section where it best fits best.

Methods

1. Unique molecular identifiers are mentioned (page 2), but UMIs are still relatively new. I suggest the authors provide a little more background information on UMIs, specifically to help clarify to readers how deduplication by UMI is distinct from standard deduplication, which is an important point the authors make in this section.

Authors' response: We added a couple of sentences to explain the UMIs, the reason behind using them, and how we deduplicate based on UMIs.

2. The colored reads in Figure 1 ("Reads (FASTQ)", "whole/split reference (FASTA)") are confusing. Read deduplication is represented (removal of a dark blue read), but the brown read disappears and then reappears in the alignment step, and there are colored reads in the alignment step that don't appear in the first step. I suggest ensuring that the representation of the method in Figure 1 is consistent with the described method.

Authors' response: We thank the reviewer for this important observation. We changed the read colors in Figure 1 and made sure that these colors are consistent throughout the figure. Additionally, we changed the representation of the "Building common read loci" and "Quantification of CRLs" steps. Now the quantification is shown as bars that are generated by the read segment counts for each CRL. We also extended the figure caption with more explanation on that particular example in the figure.

3. The section on choosing which reference to align to (page 4) would benefit from more discussion on the pros and cons of choosing the genome vs the transcriptome. The authors provide two reasons for choosing to align to the transcriptome, but it would be interesting to hear the authors' thoughts on whether aligning to the transcriptome could be affected by how well-defined an organism's transcriptome is. Especially in the context of miRNA:target mapping, where many miRNA targets are in 3'UTRs, organisms with poorly annotated transcriptomes (missing isoforms where expression is cell- or tissue- or developmental-specific) or poorly defined 3'UTRomes will potentially miss mapping (higher false negatives). These mappings could be recovered if reads are aligned to the genome. I think readers would benefit from an expansion of this section.

Authors' response: We agree that giving reasons to align to transcriptome only seems to be incomplete. We extended the text and gave reasons to map to the whole genome, as suggested by the reviewer.

Results and Discussion

1. "Based on the benchmark data provided by the CLAN publication, we produced our benchmark data to test the performance of ChiRA." (page 6). It would be great if the authors could publish or make available the benchmark data they generated and used, for example in the ChiRA GitHub repository.

Authors' response: We created a Zenodo dataset with the benchmark data (https://zenodo.org/record/4289365) and added a citation from the section "Availability of supporting data and materials".

2. "Each read is a direct fusion of (sub)sequences of human hg38 miR-Base [34] mature miRNAs and a random TargetScan [35] target sequence (i.e., the target sequence is not necessarily a true target of this miRNA)." (page 6). If the benchmark data is made of TargetScan predicted target sequences, does

this bias the benchmark data for computationally predicted interactions, which has potentially many false positives?

Authors’ response: We believe that there is no bias in this regard, as we did not optimize any parameters or built any models to guide our method. Please note that there is no relation between the fused sequences. We used benchmark data to show that our method can pick the correct alignments out of all possible multi mappings. Note that unlike CLAN benchmark data, our benchmark data contain reference sequences of varying lengths. Hence, we believe that it is a robust evaluation.

3. In Figure 4, the observation that PSI > 90% for all methods supports the authors’ conclusion. The experiment would be stronger if the authors included a baseline PSI, for example using generated random regions of the reference or some form of random sampling, like was done for Figure 5.

Authors’ response: Thank you for the suggestion. We did a random sampling of sequences and computed PSI as a baseline for Figure 4 and updated the text.

4. “Though not all of the CRLs have explainable sources (for eg, CLEAR-CLIP and SPLASH), they are far better than randomly sampled genes.” (page 8). I agree with the conclusion presented, but I suggest using objective language instead of the subjective “far better” to describe that genes from the same CRL are more often found in the same gene family or KEGG pathway than randomly selected genes.

Authors’ response: We take the suggestion from the reviewer and changed the sentence to “Although not all of the CRLs have explainable sources (for eg, CLEAR-CLIP and SPLASH), overall the genes from a CRL are more often belong to a same gene family or KEGG pathway than randomly sampled genes”.

5. “There is a large overlap of 83% with CLASH and 73% with CLEAR-CLIP published interactions despite using different aligners. Compared to the published dataset(s), ChiRA on average detects three times more interactions.” (page 9). I am wondering what the authors conclude from this result. Do they think ChiRA is producing many more false positives? Identifying more true positives than CLASH or CLEAR-CLIP?

Authors’ response: Thank you for your question. Unfortunately, there is no “ground truth” dataset to our knowledge available to test whether the detected interactions are true positives or not. From our analysis of benchmark data and supported by IntaRNA hybridization of interacting loci, it is likely that the majority of these detected interactions are true positives. We added a sentence at the end of the section “Sensitive chimeric read detection using ChiRA”.

Other

1. The Galaxy tutorial was informative and straightforward to follow. I greatly appreciate the authors making this available, and I think readers will find it very helpful. When I get to the “Viewing individual interaction information” section, step “Click on one of the records to view following information”, nothing shows up in the middle panel when I select one of the records (using Galaxy Europe). I don’t propose this is required for publication, but I suggest QA testing to ensure this visualisation works for users.

Authors’ response: To view individual interaction information, the “+” icon should be clicked which shows all the records having the same combination of gene symbols on the left panel. Clicking on one of these records will show relevant information about the selected interaction in the middle panel. Alternatively, one or more checkboxes can be selected and then clicking on the summary button will show a summary of all the selected interactions in the middle panel. We updated the training material text and the figure with highlighted details on how to view a single record.

2. Competing Interests and Authors’ information sections appear to contain boilerplate text. Either remove these sections or replace with relevant text.

Authors’ response: Thank you for pointing this out. We modified the Competing Interests section and removed the Authors’ information section.

Close
