# Supplementary material for: ChiRA: an integrated framework for chimeric read analysis from RNA-RNA interactome and RNA structurome data
Source: Gigascience. 2021 Jan 29;10(2):giaa158. doi: 10.1093/gigascience/giaa158 (PMC7844879; doi:10.1093/gigascience/giaa158)
Supplement: giaa158_Response_to_Reviewer_Comments_Revision_1 [file giaa158_response_to_reviewer_comments_revision_1.pdf]

Author's Response To Reviewer Comments

Close

Dear Nicole Nogoy,
We made changes to the manuscript as per your suggestions. Additionally, we removed the red colored highlighting added link to RRID resource.
Thank you and best regards,
Pavankumar Videm

Close
