# Supplementary material for: ChiRA: an integrated framework for chimeric read analysis from RNA-RNA interactome and RNA structurome data
Source: Gigascience. 2021 Jan 29;10(2):giaa158. doi: 10.1093/gigascience/giaa158 (PMC7844879; doi:10.1093/gigascience/giaa158)
Supplement: giaa158_Reviewer_1_Report_Original_Submission — Anil Thanki -- 9/16/2020 Reviewed [file giaa158_reviewer_1_report_original_submission.pdf]

Reviewer Report

Title: ChiRA: an integrated framework for Chimeric Read Analysis from RNA-RNA interactome and RNA structurome data

Version: Original Submission **Date:** 9/16/2020

Reviewer name: Anil Thanki

Reviewer Comments to Author:

Authors of the paper have put together an excellent workflow for RNA-interactome data analysis. Making the workflow available in Galaxy shows a great effort by authors to make it available for a wider audience. ChiRAViz is also a great addition to the work to visualise the results within Galaxy.
I am not an expert to judge the biological aspects of work, however, authors have tried their best to explain every aspect of the workflow in detail. I am reviewing the tool/software and visualisation aspects of the tool and manuscript.
Here, authors have put in a lot of effort to make their work available to a wider audience by making source code available from GitHub and installation from Conda package management system as well the whole workflow and intermediary tools as available in Galaxy public instance.
Authors also prepared Galaxy training material with test data and step by step guide to execute the workflow.
Following minor changes to manuscript and workflow will be good:
1. Authors have not provided a direct link to the workflow configuration file (.ga) for users to download and execute in local Galaxy, which will also allow tailoring workflow by swapping tools if needed. Though I managed to find workflow source code from Galaxy training material.
2. When running ChiRA, as a Galaxy workflow aligner option in ChiRAmap can only be changed in workflow editor view, it may be worthwhile to look into an option which allows changing aligner in workflow run view.
3. I have run the workflow a couple of times and the output format for SQLite database is set to .sqlite, it needs to be converted into chira.sqlite to enable visualisation plugin. The format should have been set to chira.sqlite.
4. Also, a Galaxy workflow overview image in the manuscript would be a good addition.
5. In manuscript, section 'Visualization of chimeric reads' explains the visualisation well, but intext reference to figure and legend is missing which will help the reader to navigate while reading the text.
The resulting SQLite database has one large table, I would suggest splitting the database into separate tables to make it more scalable and also potentially reusable by other tools.
Overall, the authors have put together a comprehensive framework and a well descriptive manuscript. I would like to see this manuscript published.

Level of Interest

Please indicate how interesting you found the manuscript: Choose an item.

Quality of Written English

Please indicate the quality of language in the manuscript: Choose an item.

Declaration of Competing Interests

Please complete a declaration of competing interests, considering the following questions:

- Have you in the past five years received reimbursements, fees, funding, or salary from an organisation that may in any way gain or lose financially from the publication of this manuscript, either now or in the future?
- Do you hold any stocks or shares in an organisation that may in any way gain or lose financially from the publication of this manuscript, either now or in the future?
- Do you hold or are you currently applying for any patents relating to the content of the manuscript?
- Have you received reimbursements, fees, funding, or salary from an organization that holds or has applied for patents relating to the content of the manuscript?
- Do you have any other financial competing interests?
- Do you have any non-financial competing interests in relation to this paper?

If you can answer no to all of the above, write 'I declare that I have no competing interests' below. If your reply is yes to any, please give details below.

I declare that I have no competing interests

I agree to the open peer review policy of the journal. I understand that my name will be included on my report to the authors and, if the manuscript is accepted for publication, my named report including any attachments I upload will be posted on the website along with the authors' responses. I agree for my report to be made available under an Open Access Creative Commons CC-BY license (http://creativecommons.org/licenses/by/4.0/). I understand that any comments which I do not wish to be included in my named report can be included as confidential comments to the editors, which will not be published.

Choose an item.

To further support our reviewers, we have joined with Publons, where you can gain additional credit to further highlight your hard work (see: https://publons.com/journal/530/gigascience). On publication of this paper, your review will be automatically added to Publons, you can then choose whether or not to claim your Publons credit. I understand this statement.

Yes Choose an item.
